# Defining the features and structure of neutralizing antibody targeting the silent face of the SARS‐CoV‐2 spike N‐terminal domain

**DOI:** 10.1002/mco2.70008

**Published:** 2024-11-28

**Authors:** Zhaoyong Zhang, Yuanyuan Zhang, Yuting Zhang, Linling Cheng, Lu Zhang, Qihong Yan, Xuesong Liu, Jiantao Chen, Jun Dai, Yingying Guo, Peilan Wei, Xinyi Xiong, Juxue Xiao, Airu Zhu, Jianfen Zhuo, Ruoxi Cai, Jingjun Zhang, Haiyue Rao, Bin Qu, Shengnan Zhang, Jiaxin Feng, Jinling Cheng, Jingyi Su, Canjie Chen, Shu Li, Yuanyuan Zhang, Lei Chen, Yingkang Jin, Yonghao Xu, Xiaoqing Liu, Yimin Li, Jingxian Zhao, Yanqun Wang, Qiang Zhou, Jincun Zhao

**Affiliations:** ^1^ State Key Laboratory of Respiratory Disease National Clinical Research Center for Respiratory Disease Guangzhou Institute of Respiratory Health the First Affiliated Hospital of Guangzhou Medical University Guangzhou China; ^2^ Center for Infectious Disease Research Research Center for Industries of the Future Zhejiang Key Laboratory of Structural Biology School of Life Sciences Westlake University Institute of Biology Westlake Institute for Advanced Study Westlake Laboratory of Life Sciences and Biomedicine Hangzhou Zhejiang Province China; ^3^ Health and Quarantine Laboratory Guangzhou Customs District Technology Centre Guangzhou China; ^4^ Southern University of Science and Technology Shenzhen China; ^5^ Guangzhou National Laboratory Bio‐Island Guangzhou China; ^6^ Shanghai Institute for Advanced Immunochemical Studies School of Life Science and Technology Shanghai Tech University Shanghai China; ^7^ Pediatric Pulmonary Department Guangzhou Women and Children's Medical Center Guangzhou Medical University Guangzhou China; ^8^ Clinical Laboratory Medicine Department The Second Affiliated Hospital of Guangzhou Medical University Guangzhou China; ^9^ GMU‐GIBH Joint School of Life Sciences Guangzhou Medical University Guangzhou China; ^10^ Institute for Hepatology National Clinical Research Center for Infectious Disease The Second Affiliated Hospital School of Medicine Shenzhen Third People's Hospital Southern University of Science and Technology Shenzhen China

**Keywords:** glycan shield, neutralizing antibody, N‐terminal domain, SARS‐CoV‐2, silent face

## Abstract

Research on virus/receptor interactions has uncovered various mechanisms of antibody‐mediated neutralization against severe acute respiratory syndrome coronavirus 2 (SARS‐CoV‐2). However, understanding of neutralization by antibodies targeting the silent face, which recognize epitopes on glycan shields, remains limited, and their potential protective efficacy in vivo is not well understood. This study describes a silent face neutralizing antibody, 3711, which targets a non‐supersite on the N‐terminal domain (NTD) of the spike protein. Cryo‐EM structure determination of the 3711 Fab in the spike complex reveals a novel neutralizing epitope shielded by glycans on the spike's silent face. Antibody 3711 inhibits the interaction between the receptor‐binding domain (RBD) and human angiotensin‐converting enzyme 2 (hACE2) through steric hindrance and exhibits superior in vivo protective effects compared to other reported NTD‐targeted monoclonal antibodies (mAbs). Competition assays and antibody repertoire analysis indicate the rarity of antibodies targeting the 3711‐related epitope in SARS‐CoV‐2 convalescents, suggesting the infrequency of NTD silent face‐targeted neutralizing antibodies during SARS‐CoV‐2 infection. As the first NTD silent face‐targeted neutralizing antibody against SARS‐CoV‐2, the identification of mAb 3711, with its novel neutralizing mechanism, enhances our understanding of neutralizing epitopes on glycan shields and elucidates epitope‐guided viral mutations that evade specific antibodies.

## Graphical abstract

Silent face targeted neutralizing antibodies of SARS‐CoV‐2 have been rarely reported. We described a neutralizing antibody 3711 targeting a novel epitope shielded by glycans on the spike's silent face, which inhibits the interaction between RBD and hACE2 via steric hindrance. Identification of antibody 3711 with a novel neutralizing mechanism enhances our understanding of neutralizing epitopes on glycan shields of spike.

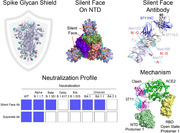

## 1 INTRODUCTION

The ongoing emergence of severe acute respiratory syndrome coronavirus 2 (SARS‐CoV‐2) variants poses significant challenges to global public health. Due to extensive mutations in the spike protein, Omicron and its sub‐lineages have gained a remarkable ability to evade immunity conferred by vaccines or previous infections. Furthermore, the immune evasion by Omicron sub‐lineages has significantly contributed to their extremely high transmission rates.^1^, ^2^ Consequently, the discovery of novel protective epitopes and mechanism analysis is urgently needed.

The SARS‐CoV‐2 spike protein is critical in the entry and fusion process, which is initiated by its interaction with human angiotensin‐converting enzyme 2 (hACE2). The spike trimer undergoes flexible conformational changes when interacting with hACE2, transitioning from the prefusion to postfusion form. Nearly 97% of spike trimers are in the prefusion form in intact virions.^3^, ^4^ Mutations highly accumulated in the Omicron spike protein significantly disrupt the neutralization capacity of receptor‐binding domain (RBD)‐directed neutralizing antibodies.^2^, ^5^ The N‐terminal domain (NTD) of the spike is an alternative target for neutralization.^6^, ^7^, ^8^ Most NTD‐directed neutralizing antibodies target an antigenic “supersite”.^9^, ^10^, ^11^ However, supersite‐targeting antibodies are particularly vulnerable to escape by variants of concern (VOCs).^12^, ^13^ Antibodies binding to distinct regions from the antigenic supersite, such as 5–7,^14^ P008_056,^15^ and DH1052,^16^ have been sporadically reported. Some of these antibodies demonstrated greater neutralization breadth against VOCs than supersite‐directed antibodies, indicating the potential value of monoclonal antibodies (mAbs) targeting the non‐supersite on the NTD for Coronavirus disease 2019 (COVID‐19) pandemic control.^8^, ^14^, ^15^, ^16^, ^17^

Similar to HIV gp120, the SARS‐CoV‐2 spike utilizes a glycan shield to evade the host immune response. However, the level of glycan shielding in the spike protein is more moderate, with about 60% of the surface potentially accessible to antibodies.^18^ The high density of glycans creates a shield that blocks antibody recognition, known as the “silent face”. The surface of the SARS‐CoV‐2 spike monomer displays 22 asparagine (Asn)‐linked glycans, with 8 located in the NTD.^19^, ^20^ The silent face is believed to exist in the highly glycan‐decorated SARS‐CoV‐2 spike NTD, which may be involved in immune evasion by impeding antibody recognition. Meanwhile, silent face‐targeted neutralizing antibodies can directly contact glycans around the silent face center, facilitating interaction with the shielded epitope.

Rare silent face targeted neutralizing antibodies of SARS‐CoV‐2 have been reported.^21^, ^22^ It remains unclear whether neutralizing antibodies targeting the NTD silent face exist and, if they do, what their defining characteristics are. It is also not clear how SARS‐CoV‐2 evade such silent face‐targeting antibodies. Herein, we identified a SARS‐CoV‐2 NTD‐targeted neutralizing antibody, designated 3711, from a SARS‐CoV‐2 convalescent. Detailed structural determination of 3711 in the spike complex revealed a novel neutralizing epitope shielded by glycans on the spike's silent face. mAb 3711 inhibits the interaction between the RBD and hACE2 via steric hindrance. Identifying antibodies targeting this silent face would be beneficial for fostering a systematic understanding of antibody epitopes on SARS‐CoV‐2.

## 2 RESULTS

### 2.1 Characteristics and overall conformation of two SARS‐CoV‐2 NTD‐targeted neutralizing antibodies

**FIGURE 1:**
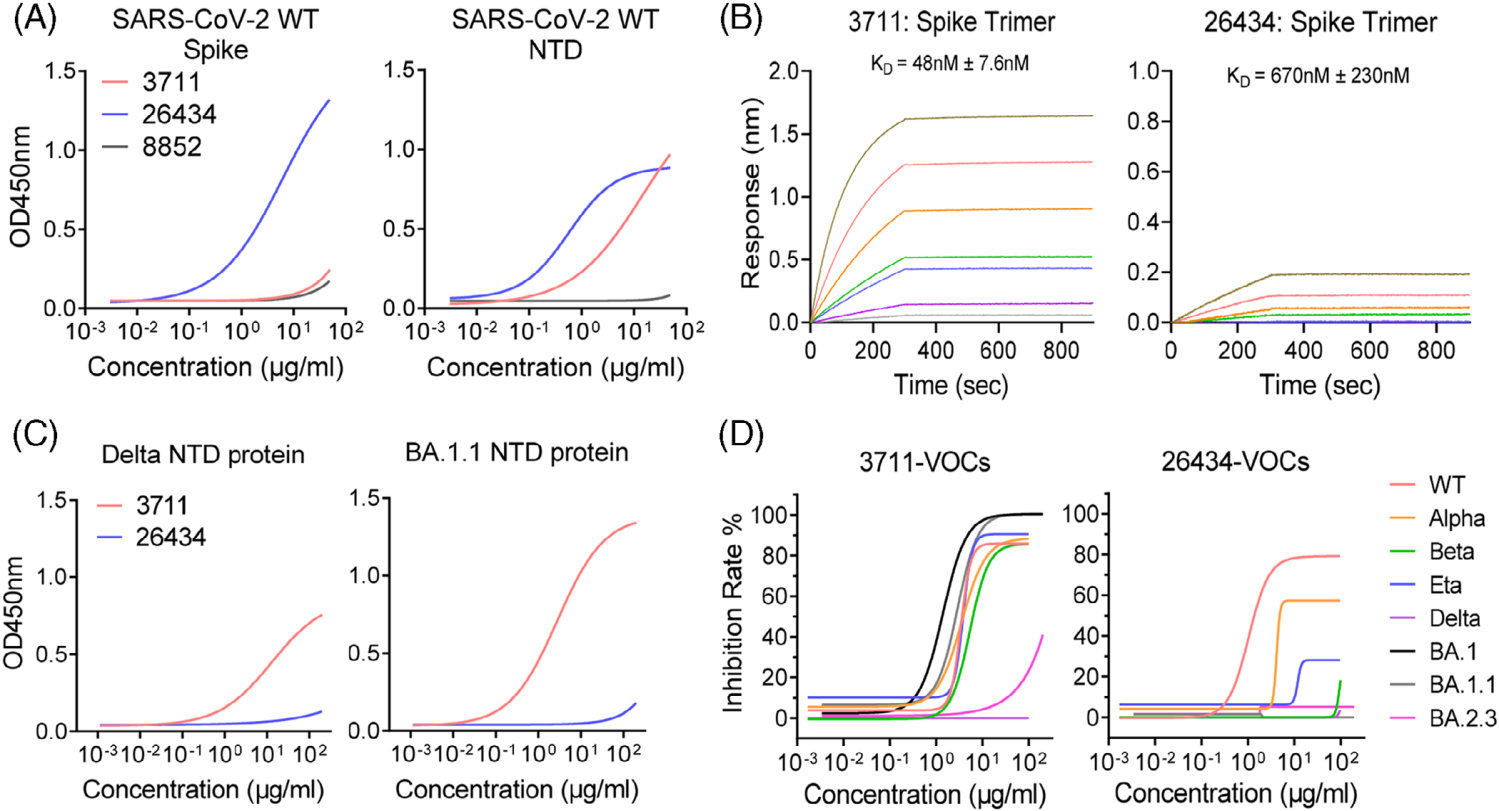
Characteristics of two antibodies targeting severe acute respiratory syndrome coronavirus 2 (SARS‐CoV‐2) spike N‐terminal domain. (A) ELISA (enzyme‐linked immunosorbent assay) binding characteristic of mAb 3711 and 26,434 to SARS‐CoV‐2 S1+S2 extracellular domain (ECD)–His recombinant and N‐terminal domain (NTD) proteins. Three independent experiments were performed, and one representative result is shown. Spike protein and NTD protein were from Sino Biological (40589‐V08B1) and Novoprotein (DRA45), respectively. (B) The binding affinity of mAb 3711 and 26,434 to SARS‐CoV‐2 Spike by biolayer interferometry (BLI). Representative results of two replicates are shown. (C) ELISA binding characteristic of mAb 3711 and 26,434 to NTD protein of SARS‐CoV‐2 Delta and BA.1.1. (D) Neutralization capacity of mAb 3711 and 26,434 to SARS‐CoV‐2 authentic virus by focus reduction neutralization test (FRNT assay). Data are representative of results among three independent experiments. VOCs, variants of concern.

To comprehensively profile the NTD‐targeting neutralizing antibodies (nAbs) induced in COVID‐19 convalescents, peripheral blood mononuclear cells (PBMCs) from COVID‐19 convalescents infected with SARS‐CoV‐2 wild‐type (WT) Wuhan‐Hu‐1, exhibiting robust specific antigen responses, were used for neutralizing antibody screening via Epstein‐Barr virus (EBV) immortalization.^23^ Two SARS‐CoV‐2 NTD‐targeted nAbs, designated as 3711 and 26,434, were identified. Antibody 3711, encoded by IGHV3‐11 and IGKV1‐17, with a relatively long CDRH3 of 19 amino acids, binds exclusively to the SARS‐CoV‐2 NTD protein, rather than the spike monomer protein. In contrast, antibody 26,434, encoded by IGHV1‐18 and IGLV3‐1, with a moderately long CDRH3 of 13 amino acids, binds both the NTD protein and the spike monomer protein. The two NTD‐targeting antibodies exhibited distinct binding patterns (Figure 1A). Biolayer interferometry (BLI) revealed that antibodies 3711 and 26,434 bind to the SARS‐CoV‐2 WT spike trimer protein with affinities of 48 and 670 nM, respectively (Figure 1B). Antigen‐binding specificity analysis indicated that antibody 26,434 loses its binding capacity to Delta (B.1.617.2) and Omicron (B.1.1.529) NTD proteins, whereas antibody 3711 retains potent binding to Omicron NTD protein, with partially reduced binding to Delta NTD protein (Figure 1C).

To evaluate their neutralizing activities against SARS‐CoV‐2 variants, authentic virus neutralization assays were conducted in a BSL‐3 facility. Antibody 3711 effectively neutralized WT, Alpha, Beta, Eta, and Omicron BA.1.1 strains with EC_50_ values of 3.388 μg/mL, 3.531 μg/mL, 5.317 μg/mL, 3.827 μg/mL, and 3.376 μg/mL, respectively (Figure 1D). In contrast, mAb 26,434 failed to neutralize the Beta, Eta, Delta, and Omicron BA.1.1 strains. Specifically, all NTD‐targeted antibodies, including mAb 3711 and 26,434, were ineffective against the latest JN.1 strain.

### 2.2 Antibody 3711 targeted a cryptic epitope on the NTD silent face and directly interacts with N‐linked glycans

**FIGURE 2:**
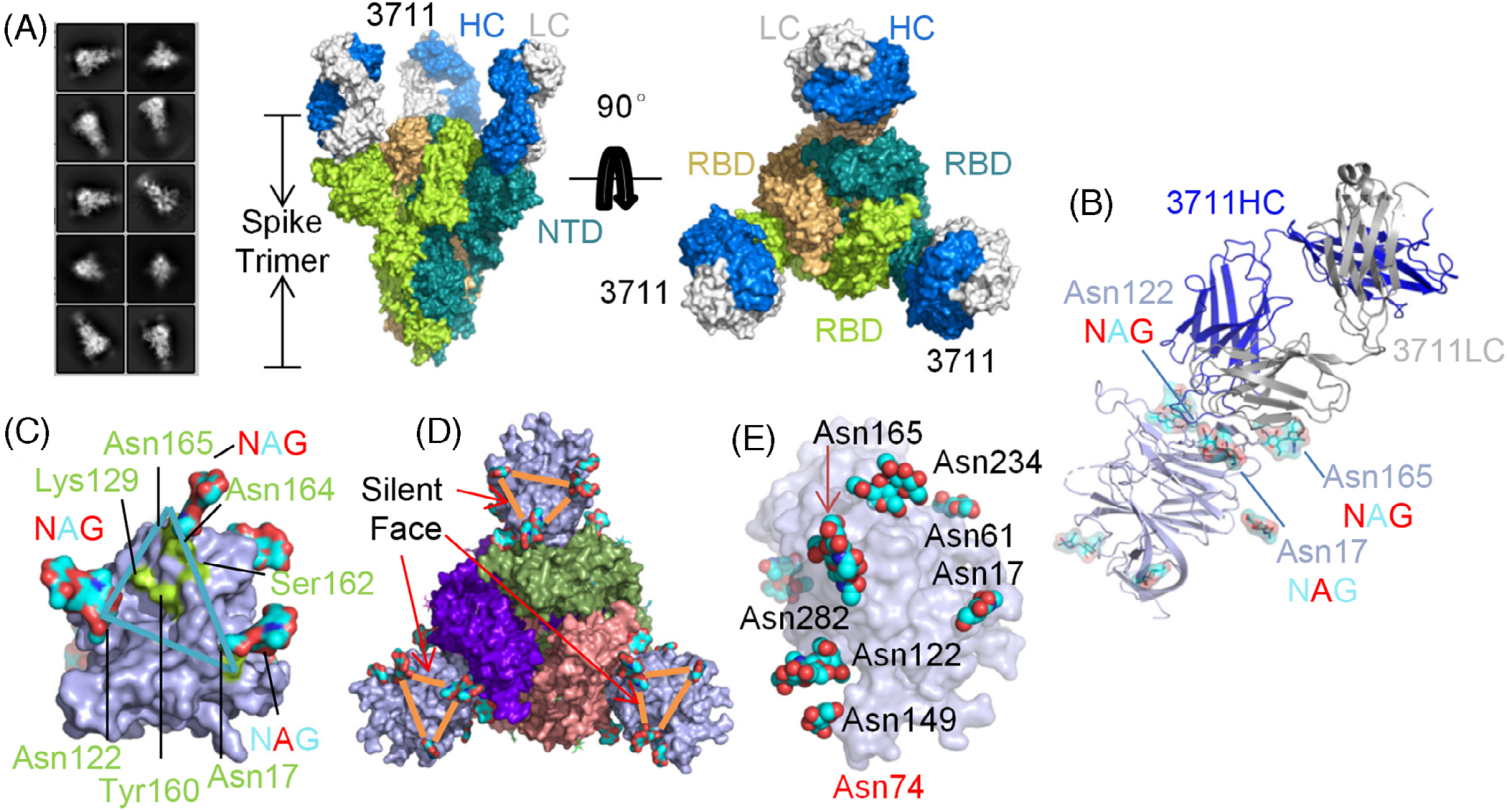
Overall conformation of severe acute respiratory syndrome coronavirus 2 (SARS‐CoV‐2) spike N‐terminal domain “silent face” antibody 3711. (A) 2D classification, modeling, and Cryo‐EM map showing the surface of mAb 3711 in complex with SARS‐CoV‐2 spike extracellular domain (ECD) with two perpendicular views. The heavy chain and light chains of 3711 are colored blue and gray, respectively. The monomers of trimer spike protein (all receptor‐binding domains [RBDs] are in a closed state) are colored deep teal, limon, and wheat, respectively. (B) The overall conformation of mAb 3711 in complex with SARS‐CoV‐3 N‐terminal domain (NTD) in cartoon type. SARS‐CoV‐2 spike NTD is colored light blue in surface type; asparagine‐linked glycans are represented as cyan, blue, and red in spheres. (C) An epitope surrounded by Asn17‐, Asn122‐ and Asn165‐ linked glycans on NTD protein was designated as a “silent face”. Asparagine‐linked glycans are shown in cyan, blue, and red; the footprint of 3711 interacting as NTD is colored in limon. (D) The location of NTD “silent face” on trimer spike protein (PDB: 7C2L). Spike with three RBDs “down” is shown in the top view. NTD is colored light blue; three RBDs are shown in purple, smudge, and salmon, respectively. (E) Overall conformation as a surface type of SARS‐CoV‐2 NTD protein (PDB: 7C2L) with high glycosylation. Asparagine‐linked glycans are represented as cyan and red spheres. Asn74 linked glycans were undetected in 7C2L model. All protein structures were processed in PyMOL. NAG, N‐acetylglucosamine (GlcNAc).

To characterize the epitopes of mAbs 3711 and 26434, we solved the Cryo‐EM structures of 3711 and 26,434 in complex with the SARS‐CoV‐2 (WT) spike‐ECD to determine their overall conformations (Figures S1 and S2). According to 2D classification analysis, antibody 3711 binds exclusively to the spike in the prefusion conformation with all three RBDs in the down conformation (Figure 2A). Each spike trimer was bound to three 3711 Fabs symmetrically, highlighting a specific antibody‐spike conformation. Detailed interaction mapping of NTD‐3711 revealed a cryptic epitope surrounded by three N‐linked glycans (Asn17‐NAG, Asn122‐NAG, and Asn165‐NAG), involving 37 amino acids, which form a flat face on each NTD of the Spike trimer (Figure 2B–D). The N‐terminal domain (NTD) of the SARS‐CoV‐2 Spike protein is highly glycosylated, occupying 36.4% (8 out of 22) of the Asn‐linked glycan sites on the spike, while accounting for 22.9% (292 out of 1273) of the total spike protein (Figure 2E). The concept of epitopes shielded by glycans and devoid of antibody recognition, known as the “silent face”, has been reported in HIV gp120 but not yet for SARS‐CoV‐2 Spike NTD protein.^24^, ^25^

**FIGURE 3:**
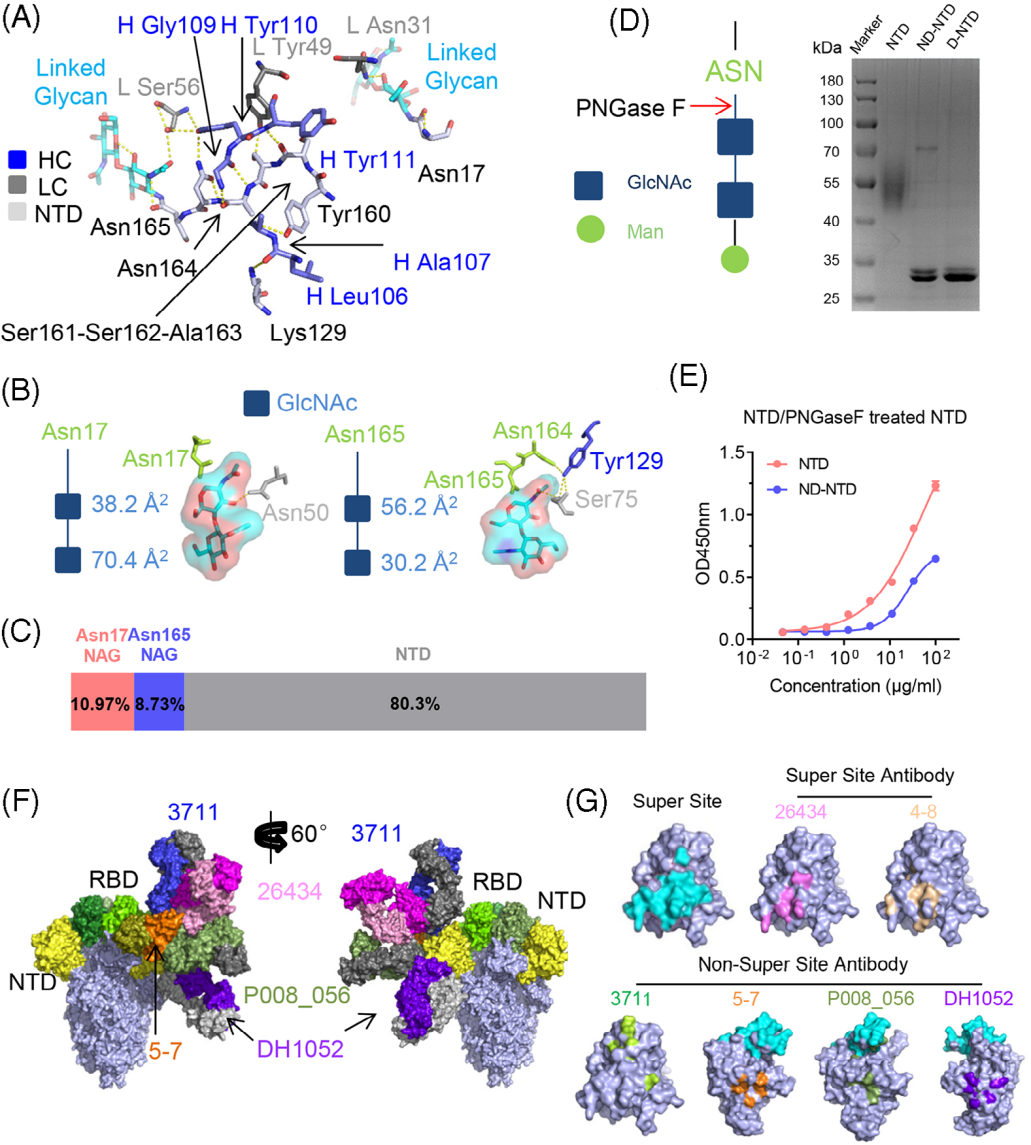
mAb 3711 directly interacts with Asn‐linked glycans and shows a distinct epitope footprint. (A) Detailed interactions between mAb 3711 and severe acute respiratory syndrome coronavirus 2 (SARS‐CoV‐2) NTD. The residues involved in interactions are shown as sticks. The residues of the heavy chain and light chain of 3711 are colored blue and gray, respectively, and the residues of NTD are colored in light blue; Asn17 and Asn165‐linked glycans on NTD interacting with 3711 are colored in cyan. Yellow dashed lines indicate polar interactions. (B and C) Total area and proportion of NTD Asn17 and Asn165 linked glycans interacting with antibody 3711, accounting for the total buried surface area (BSA) of the complex of antibody 3711 and NTD. The model of Asn‐linked GlcNAc is colored in dark blue; Asn17 and Asn165 are colored in limon; GlcNAcs are colored in cyan and red in the type of sticks with surface shadow. The values of BSA are calculated by PDBePISA (EMBL‐EBI). (D) SDS‐PAGE analysis of deglycosylated NTD. A simple model of asparagine‐linked glycosylation and glycans digestion of PNGase F is presented on the left. The extent of deglycosylation is assessed by mobility shifts. (E) Binding capacity of antibody 3711 to deglycosylated NTD was reduced compared to untreated NTD. A protein binding assay was presented. (F) Published NTD targeting antibodies were superposed on one monomer of spike trimer (PDB: 6VXX) with two side views, including P008_056 (PDB: 7NTC), 5–7 (PDB: 7RW2), DH1052 (PDB: 7LAB) as well as 26,434 introduced in this study. The heavy chain (HC) and light chain (LC) of 3711 are colored blue and gray, respectively (26,434 HC and LC, dark pink and light pink; P008_056 HC and LC, smudge and gray; DH1052 HC and LC, dark purple and gray; 4–7 HC and LC, orange and deep olive). NTDs are colored in yellow. (G) The epitope footprint of published antibodies to NTD. The designated NTD “supersite” is indicated in cyan. The footprints of 26,434, 4–8 (PDB: 7LQV), 3711, 5–7, P008_056, as well as DH1052 are colored in pink, sand, limon, orange, olive, and purple, respectively. All protein structures were processed in PyMOL. D‐NTD, denaturing NTD digested by PNGase F; GlcNAc, N‐acetylglucosamine; Man, Mannose; NTD, naïve NTD protein without PNGase F digestion; ND‐NTD, non‐denaturing NTD digested by PNGase F.

Most NTD‐targeting neutralizing antibodies reported to date bind an epitope referred to as the “supersite”.^6^ A comprehensive analysis of NTD glycosylation profiles indicated that 3711 targets the glycans shielding or silent face (Figure S3). Key residues on the heavy chain complementarity‐determining region 3 (HCDR3) (Leu106, Ala107, Gly109, Tyr110, and Tyr111) establish polar contacts with Lys129, Tyr160, Ala163, Asn164, and Ser161 on the NTD, respectively. Additionally, Asn31 on the light chain CDR1 (LCDR1) and Tyr49 on LC framework region 2 (FR2) form polar contacts with Asn17‐linked glycan and Ser162, respectively. Ser56 on LC FR3 interacts with the NTD Asn165‐linked glycan, facilitated by Tyr110 on HCDR3, ultimately forming an intricate contact between the NTD, the heavy chain, and the light chain (Figure 3A and Figure S3).

Further detailed analysis of the antigen‐antibody interaction based on the cryo‐EM structure of NTD‐3711 revealed a novel interaction pattern. Our findings show that 3711 binds to the top flank of the NTD, engaging residues Lys129, Tyr160, Ser162, and Asn164, along with Asn164‐Asn165‐linked glycans, all situated on the NTD silent face (Figures 2C and 3A). In our structural model, both Asn17‐linked and Asn164‐Asn165‐linked glycans exhibit two N‐acetylglucosamine (GlcNAc) units. The proximal GlcNAc of Asn17 interacts with 3711 Light Chain Asn31, contributing 3.86% (38.2 Å^2^) of the total buried surface area (BSA), while the distal GlcNAc contributes 7.11% (70.4 Å^2^) of total BSA. For Asn164‐Asn165‐linked GlcNAcs, the proximal one directly interacts with 3711 heavy chain Tyr110 and light chain Ser56, covering 5.68% (56.2 Å^2^) of total BSA, whereas the distal one contributes 3.05% (30.2 Å^2^) (Figure 3B,C). The glycan‐3711 interaction (Asn17 NAG + Asn165 NAG) occupies 19.7% of the total interaction area, underscoring its pivotal role in antigen‐antibody recognition and neutralization (Figure 3B,C).

To assess the glycosylation status of NTD and its impact on 3711 binding, native purified NTD protein was incubated with PNGase F, an enzyme that cleaves all N‐linked carbohydrate moieties. The predicted molecular weight of the SARS‐CoV‐2 NTD protein is approximately 35 kDa, but it migrates as a 40–70 kDa band in SDS‐PAGE under reducing conditions due to complex glycosylation. After PNGase F treatment, SDS‐PAGE analysis showed the expected reduction in molecular mass to nearly 35 kDa (Figure 3D). To further assess the reactivity of glycan‐deleted NTD protein to antibody 3711, we tested the binding of antibody 3711 to PNGase F‐treated NTD protein. Compared to the untreated NTD protein, 3711 exhibited a significant reduction in binding capacity to the PNGase F‐treated NTD, demonstrating the glycan‐dependent binding of 3711 (Figure 3E).

Several NTD targeting antibodies have been previously reported, including “supersite” antibodies [4–8 (PDB: 7LQV)] and “non‐supersite” antibodies [5–7 (PDB: 7RW2), P008_056 (PDB: 7NTC) and DH1052 (PDB: 7LAB)] (Table S2).^11^, ^14^, ^15^, ^16^ Notably, the “NTD supersite” epitope spans regions N1 (residues 14–26), N3 (residues 141–156), and N5 (residues 246–260) loops.^11^ To further characterize the silent face‐targeted mAb 3711, we also solved the cryo‐EM structure of mAb 26434. This antibody binds to the spike in both RBD “up” and “down” states and exhibits a binding pattern similar to previously reported supersite antibodies (Figure 3F and G). Antibody HCDR2, HCDR3, LCDR1, LCDR2, and LCDR3 were found to be involved in 26434‐NTD recognition (Figure S4). While the epitope of 3711 differs significantly from previously reported NTD‐targeting antibodies, as illustrated by the superimposition of structures 3711, 26,434, 5–7, P008_056, and DH1052 onto one protomer (Figure 3F,G).

### 2.3 ǀPotential mechanism of silent face‐targeted mAb 3711‐mediated neutralization

**FIGURE 4:**
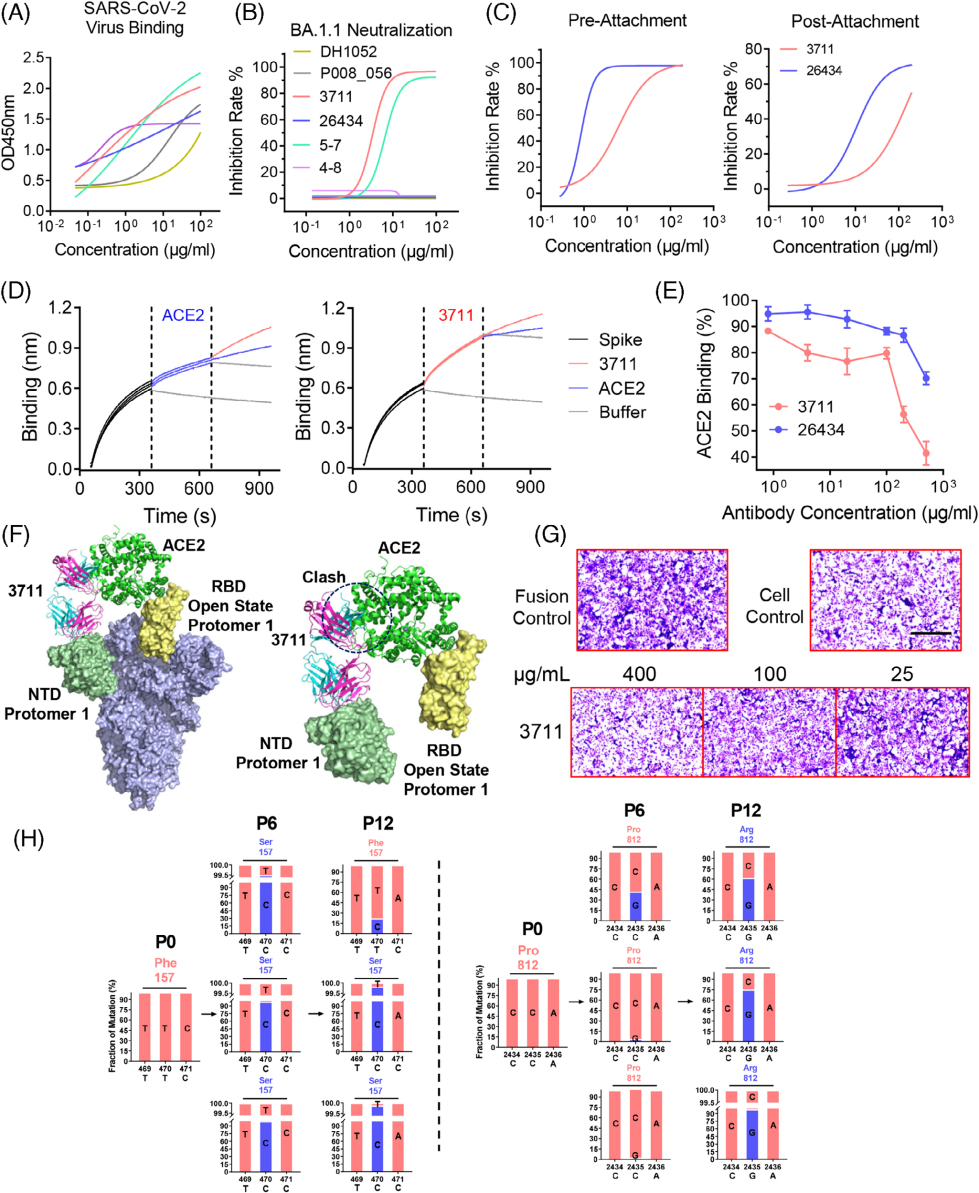
Antibody 3711 inhibits virus entry at the pre‐attachment step by preventing receptor‐binding domain (RBD)‐human angiotensin‐converting enzyme 2 (hACE2) contact. (A) Fixed severe acute respiratory syndrome coronavirus (SARS‐CoV) virion (WT) binding activity of representative NTD‐targeted antibodies. (B) Neutralization capacity of representative NTD‐targeted antibodies (DH1052, P008_056, 3711, 26434, 5–7 and 4–8) against BA.1.1. Three independent experiments were performed, and one representative result is shown. (C) Pre‐attachment (before virus attached on cell) or post‐attachment (after virus attached on cell) inhibition capacity of 3711 and 26,434 against SARS‐CoV‐2 WT strain on Vero E6 cell. Experiments were performed with technical triplicates. (D) mAb 3711 competed with hACE2 to bind to SARS‐CoV‐2 spike protein in biolayer interferometry assay. SARS‐CoV‐2 spike protein was immobilized on a streptavidin (SA) biosensor, and then hACE2 and 3711 were loaded for binding and competition, respectively. Binding signals were read by Fortebio Octet RED96e. (E) mAb 3711 competed with hACE2 to bind to SARS‐CoV‐2 (WT) Spike protein in flow cytometry analysis. SARS‐CoV‐2 spike expressing cells (HEK293T‐spike) were first incubated with antibodies 3711 and 26,434 and then with protein of hACE2 fused with mouse immunoglobulin fragment (hACE2‐mIg). The percentage of hACE2 binding corresponded to the positive rate of Anti‐Mouse IgG binding. Data are shown as mean ± Standard error of the mean (SEM) of triplicates. (F) Superposed model of spike trimer with one RBD “up” (PDB: 6VYB), antibody 3711 in complex with NTD and ACE2 in complex with RBD. The heavy and light chains of 3711 are colored blue and pink, respectively. NTD interacting with 3711 is colored in palegreen, “up” RBD is colored yellow, and ACE2 is colored in green. The clash between 3711 and ACE2 is indicated by a black dashed circle. All protein structures were processed in PyMOL. (G) Cell‐cell fusion inhibition capacity of antibody 3711. HEK‐293T cells expressing SARS‐CoV‐2 (WT) spike were coincubated with serially diluted antibody 3711 (400, 100, 25 μg/mL) and then cultured with Huh‐7 cells for further fusion; 24 hours later, cells were fixed with 4% PFA and then stained with crystal violet. Syncytia are stained in dark purple, while normal cells are colored in light purple. Scale bars (black), 200 μm. (H) Schematic for identification of antibody escaping mutants. SARS‐CoV‐2 was passaged up to 12 times in the Vero E6 cell line in the presence of serially diluted antibody 3711 (P1–P3, 1×IC_50_ of antibody 3711; P4–P6, 2×IC_50_ of antibody 3711; P7–P9, 4×IC_50_ of antibody 3711; P10–P12, 6×IC_50_ of antibody 3711). The supernatant was harvested and used for the next passage if > 50% CPE was observed. Viruses from every sixth passage (P0, P6, P12) were subject to next‐generation sequencing. The mutation screening was performed in technical triplicates. P1, passage 1. Specific escape mutations of 3711. The data of next‐generation sequencing of SARS‐CoV‐2 genome were analyzed on CLC Genomics Workbench v11.0. Two significant mutations were identified and presented in the independent triplicates of passage.

To investigate mAb 3711′s neutralization mechanism, we expressed and compared four representative NTD‐targeted neutralizing antibodies (5–7, DH1052, P008_056, and 4–8) with resolved structural information. Virion binding was assessed by fixing SARS‐CoV‐2 (WT) infected Vero E6 cells with 4% paraformaldehyde for subsequent OD_450_ recording via ELISA. Authentic virus neutralization assays indicated comparable binding of all tested antibodies (Figure 4A). However, only antibodies 3711 and 5–7 maintained neutralizing capacity against Omicron BA.1.1 (Figure 4B). We also compared the neutralization potency of 3711 and 26,434 when added before or after virus absorption to Vero E6 cells. As shown in Figure 4C, 3711 and 26,434 neutralized SARS‐CoV‐2 more efficiently when administered at a pre‐attachment step. In contrast, the potency was reduced when added post‐attachment, especially for 3711, which could not effectively neutralize SARS‐CoV‐2 at the post‐attachment step. These attachment neutralization assays demonstrated that 3711 may exert its neutralization effect at an early stage of SARS‐CoV‐2 invasion.

BLI assays were conducted to elucidate the interaction between hACE2, NTD, RBD, and mAb 3711. Figure 4D demonstrates mAb 3711′s higher affinity for SARS‐CoV‐2 (WT) spike protein compared to hACE2, competitively binding with hACE2 in BLI assays, thus interfering with hACE2‐RBD interaction. Moreover, mAb 3711 inhibited hACE2 binding to Spike in a concentration‐dependent manner (Figure 4E). Superimposing the NTD‐3711 antibody complex onto the pre‐fusion “open” state RBD revealed steric hindrance between 3711 Fab and hACE2 on one Spike protomer, blocking hACE2 binding (Figure 4F). Furthermore, mAb 3711 significantly reduced syncytia formation in cell‐cell fusion tests, similar to other NTD‐targeted antibodies (Figure 4G, Figure S5).^26^, ^27^ Overall, these results indicate that mAb 3711 functions through multiple mechanisms, including steric hindrance of RBD‐hACE2 interaction and fusion inhibition.

mAb 3711 exhibited broad neutralization against SARS‐CoV‐2 WT, Alpha, Beta, Eta, and Omicron BA.1.1 variants, but not against Delta, BA.2.3, and BA.5. Analysis of NTD mutation sites among the variants identified three mutated hotspots in Delta, BA.2.3, and BA.5, marked in red dashed boxes in Figure S6. Two of these hotspots likely contribute to immune evasion in Delta and BA.2.3 variants.^28^ The first hotspot involves T19R or T19I mutation, disrupting the Asn‐linked glycosylation motif (Asn‐X‐Ser/Thr) and resulting in loss of the 17Asn‐18Leu‐19Thr glycan. The second hotspot comprises the E156G and DEL157/158 mutations, which reshape the 143–154 loop and alter the NTD surface structure when the Delta NTD is superimposed onto the WT NTD.^13^ This reshaping affects the “silent face,” causing a clash between 3711 and the NTD, thereby impairing antibody recognition (Figures S7 and S8).

To identify critical residues for 3711 neutralization we subjected SARS‐CoV‐2 to in vitro selection of escape mutants through serial passage in the presence of serially diluted antibody 3711. Sequencing of viruses from passages 6 and 12 provided consensus sequences for alignment analysis. Notably, a unique F157S mutation emerged at passage 6 (Figure 4H), similar to mutations observed in Delta and BA.2.3 variants. Additionally, the dominant P812R mutation appeared at passage 12, located in the S2' site, which triggers spike rearrangement and virus‐host membrane fusion, potentially influenced by RBD‐hACE2 recognition.

### 2.4 mAb 3711 protects mice from SARS‐CoV‐2 WT, Beta and Omicron BA.1.1 infections

**FIGURE 5:**
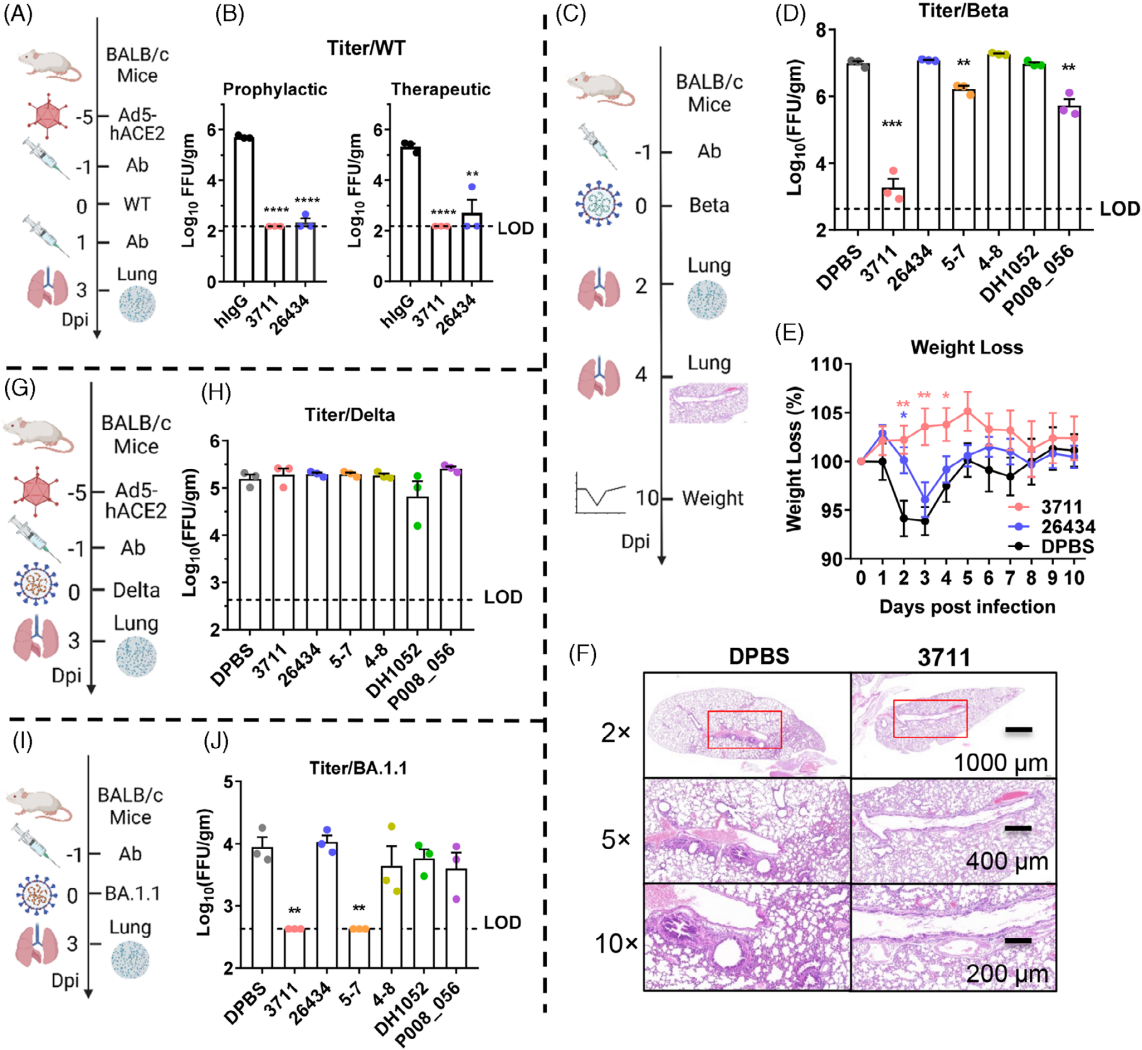
Antibody 3711 protects against severe acute respiratory syndrome coronavirus 2 (SARS‐CoV‐2) WT, Beta, and Omicron BA.1.1 challenge. (A) Overview of SARS‐CoV‐2 WT strain challenge experiment. Five days post‐transduced with 2.5×10^8^ FFU of Ad5‐hACE2, SPF BALB/c mice were challenged with 1 × 10^5^ FFU of SARS‐CoV‐2 WT. Mice were administrated with antibodies (10 mg/kg) one day before (prophylactic) or after (therapeutic) challenge. Lungs were harvested for virus tittering at 3 days post‐challenge. (B) Effect of antibody on lung virus titer with WT strain infection. N = 3 per group. LOD, limitation of detection. (C) Overview of SARS‐CoV‐2 Beta challenge experiment. Mice pre‐administrated with 10 mg/kg of antibody were challenged with 5 × 10^4^ FFU of SARS‐CoV‐2 Beta. Lungs were harvested for viral titering at dpi 2 and for pathological histology analysis (hematoxylin‐eosin staining) at dpi 4, respectively. Weight loss of mice was monitored up to 10 days post‐challenge. (D) Effect of NTD targeted antibodies on lung virus titer in the prophylactic group with Beta infection. N = 3 per group. (E) Effect of mAb 3711 on weight loss in a prophylactic group with Beta infection. N = 5 per group. (F) Effect of mAb 3711 on lung histopathological lesion upon Beta infection. N = 3 per group. Representative pathological images are shown. (G) Overview of SARS‐CoV‐2 Delta challenge experiment. Five days post‐transduced with 2.5×10^8^ FFU of Ad5‐hACE2, BALB/c mice were challenged with 5×10^4^ FFU of Delta. Mice pre‐treated with 10 mg/kg of antibody were euthanized at 3 days post‐infection for lung virus titering. (H) Effect of NTD antibodies on lung virus titer in a prophylactic group with Delta strain infection. N = 3 per group. (I) Overview of SARS‐CoV‐2 Omicron BA.1.1 challenge experiment. Mice pre‐administrated with 10 mg/kg of antibody were challenged with 5 × 10^4^ FFU of Omicron BA.1.1. Lungs were harvested for virus titering 1 day post‐challenge. (J) Effect of NTD antibodies on lung virus titer in a prophylactic group with Omicron BA1.1 infection. N = 3 per group. Schematic diagrams in Figure 5A, C, G and I are created with BioRender.com.

To assess the in vivo efficacy of silent face‐targeted antibody 3711, we employed the Ad5‐hACE2 transduced mice model for SARS‐CoV‐2 WT strain and Delta variant infections, and the BALB/c mouse model for Beta and Omicron BA.1.1 infections,^29^, ^30^ as outlined in Figure 5. Both prophylactic (one day prechallenge) and therapeutic (one day postchallenge at 10 mg/kg) administrations of mAbs 3711 and 26,434 significantly reduced lung virus titers in SARS‐CoV‐2 WT‐infected mice (Figure 5A,B). Notably, prophylactic administration of antibody 3711 more effectively prevented weight loss in SARS‐CoV‐2 Beta‐infected mice compared to mAb 26,434 (Figure 5C–E).

For an overall evaluation of the protective efficacy of targeted NTD antibodies, antibodies 5–7, 4–8, DH1052, and P008_056 were also included. Consistent with in vitro results, supersite antibodies 26434 and 4–8 showed reduced neutralization efficacy against Beta, Delta, and Omicron BA.1.1 variants. mAb P008_056 provided significant protection against Beta variant infection but not against Delta or Omicron BA.1.1 infections, whereas mAbs 3711 and 5–7 protected against Beta and Omicron BA.1.1 variants (Figure 5D,H,J). Notably, mAbs 3711, 26,434, and other NTD antibodies failed to protect mice from Delta infection, consistent with reports of Delta's evasion of most NTD‐neutralizing antibodies due to structural changes. Histological analysis of lung sections stained with hematoxylin and eosin indicated that antibody 3711 preserved alveolar structure and prevented perivascular and parenchymal infiltration (Figure 5F). Overall, these findings highlight mAb 3711′s superior in vivo protective efficacy compared to other NTD‐targeted antibodies, including supersite and non‐supersite variants.

### 2.5 3711‐Class antibody presence in a minority of COVID‐19 convalescents and its low gene usage rate

**FIGURE 6:**
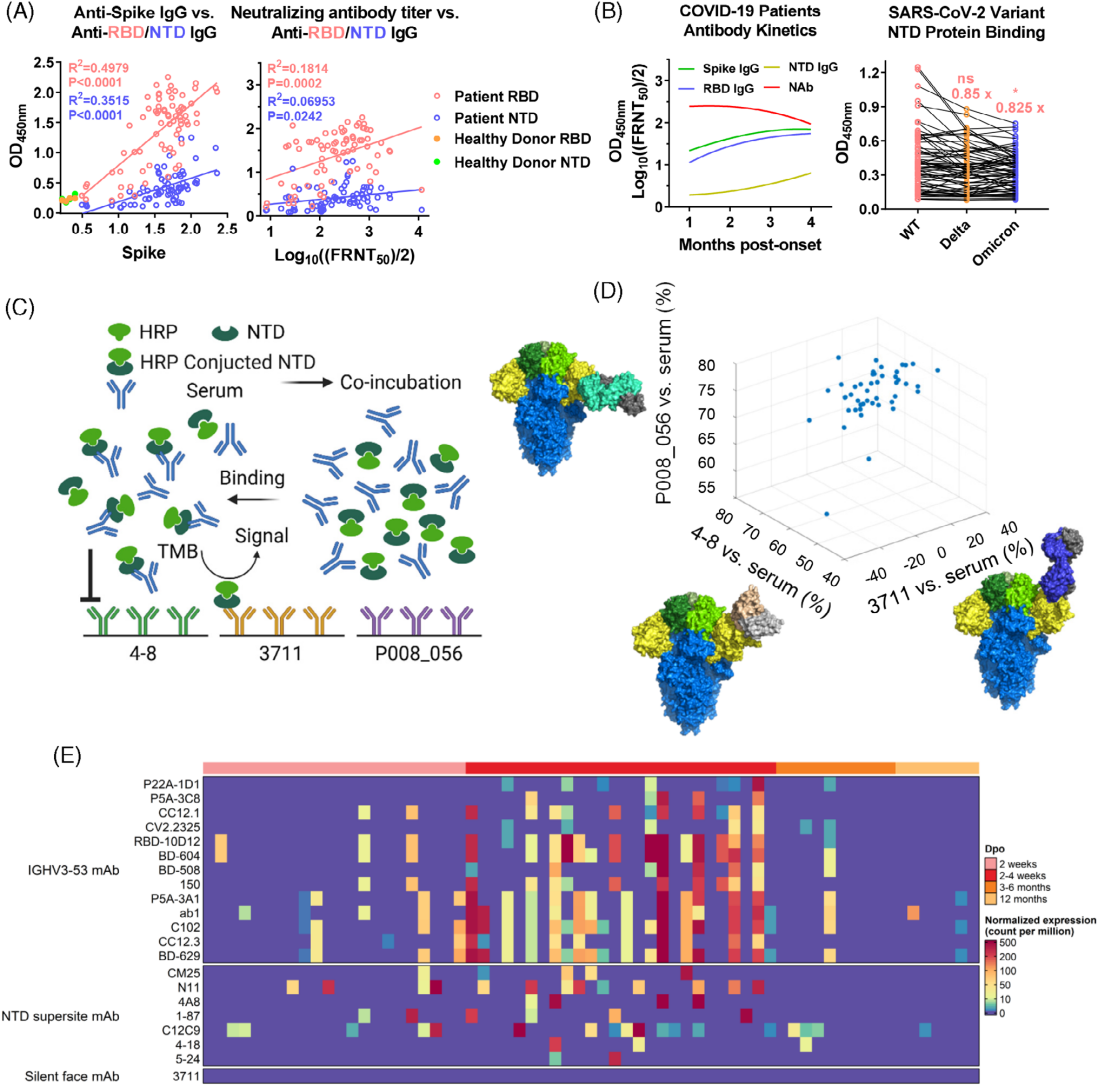
Class analysis of NTD‐specific antibodies in sera of Coronavirus disease 2019 (COVID‐19) patients. (A) Both receptor‐binding domain (RBD) and N‐terminal domain (NTD)‐specific IgGs were linearly correlated with spike‐specific IgG and neutralizing antibody titer on the sera from COVID‐19 patients infected WT in 2020. Sera were heat‐inactivated and diluted to perform a protein binding assay (ELISA) and neutralization assay (FRNT_50_). Pearson's correlation coefficient was accessed to evaluate the relationship between RBD, NTD, spike, and neutralizing antibody titer. (B) Kinetics and potency of IgG and neutralizing antibody of COVID‐19 patient. Serial serum samples of COVID‐19 patients infected with SARS‐CoV‐2 WT were collected for kinetic detection. Moreover, sera were diluted to perform a protein binding assay against NTD of WT, Delta, and Omicron BA.1.1. (C) Schematic diagram for class analysis of NTD‐specific antibody of COVID‐19 patient. The serum of patients was co‐incubated with HRP conjugated NTD, then added to an ELISA plate pre‐coated with NTD antibodies with overlapped epitopes individually (3711; 4–8, PDB: 7LQV; P008_056, PDB: 7NTC). The reaction was visualized by TMB substrate. HRP‐NTD binds to respective coated antibodies were set as negative controls. The OD_450_ value of sera < 50% of OD_450_ of HRP‐NTD binds to respective antibody was considered as an evident competition effect. Schematic diagram is drawn in Biorender. (D) Class analysis of NTD‐specific antibody of COVID‐19 patients. The *x*, *y*, and *z* axes corresponded to the competition rate between serum and antibody 4–8, 3711, and P008_056, respectively. Data were plotted and presented on Matlab. Structures of 4–8, 3711, and P008_05 were processed in PyMOL. (E) Heatmap of the abundance of antibody gene usage in antibody repertoire sequencing of 33 COVID‐19 convalescent with sequential sampling. The top annotation represents the sampling time (Dpo: Days post symptom onset). The color bar represents the normalized expression level of IgH sequences of enrolled antibodies. HRP, horseradish peroxidase; TMB, tetramethylbenzidine.

To investigate the NTD‐specific humoral response, we conducted a series of serological tests on COVID‐19 patients using SARS‐CoV‐2‐specific antigens. As illustrated in Figure 6A, NTD‐specific IgG levels correlated significantly with Spike‐specific IgG, RBD‐specific IgG, and neutralization titers, underscoring the NTD as a crucial epitope for neutralizing antibodies. Notably, neutralizing antibody titers declined at 2 months post‐onset, whereas spike and RBD‐specific IgG levels remained elevated until 4 months post‐onset. Conversely, NTD‐specific IgG levels showed an increasing trend, suggesting their prolonged presence (Figure 6B). Additionally, we assessed serum binding capacity against NTD proteins from different variants (WT, Delta, and Omicron). We observed significant reductions in Delta and Omicron BA.1 NTD binding to sera from SARS‐CoV‐2 WT‐infected patients (0.85 times and 0.825 times, respectively), indicating decreased binding of NTD‐targeted antibodies under selective pressure from mutated variants and host immune responses.

Given that the majority of reported NTD‐neutralizing antibodies target the “supersite” and the epitope of antibody 3711 remains uncharacterized, we conducted a serum‐NTD antibody competition assay to assess the distribution of the 3711‐class antibody in infected patients (Figure 6C). “Supersite site” antibody 4–8, “non‐Supersite antibody” P008_056 and “Silent Face” antibody 3711 were enrolled for detection. The results indicate that nearly all enrolled sera showed significant competition with fixed antibody 4–8 (45/46) and P008_056 (46/46), indicating widespread presence of 4–8‐like and P008_056‐like antibodies in infected patients' sera. In contrast, antibody 3711 showed minimal competition (0/46), suggesting a narrower distribution compared to 4–8 and P008_056 (Figure 6D). Therefore, compared to antibodies 4–8 and P008_056, the distribution of the 3711‐class antibody in the human population is relatively narrow. To further determine the occurrence and gene usage of the 3711‐class antibody, we summarized antibody 3711 gene data from an antibody repertoire sequencing study of 33 COVID‐19 patients with sequential sampling, as previously described.^31^, ^32^ Germline IGHV3‐53‐encoded antibodies, commonly present in COVID‐19 patients, were used as controls in the present study. As shown in Figure 6E, unlike the germline IGHV3‐53‐encoded antibodies and NTD supersite antibodies, whose normalized expression is robust, the usage of the NTD Silent Face antibody 3711 gene is consistently rare during SARS‐CoV‐2 infection. The analysis of the occurrence and prevalence of the 3711‐class antibody in antibody repertoire supports the same conclusion as the serological tests above.

## 3 DISCUSSION

Due to extensive asparagine (Asn)‐linked glycosylation, the SARS‐CoV‐2 Spike‐NTD is shielded from immune recognition, limiting the characterization of NTD‐specific neutralizing antibodies.^19^ These potential epitopes are masked by N‐glycans, creating a “silent face” that hinders B cell recognition and the induction of a robust humoral response against these shielded epitopes. The concept of the "silent face" was initially elucidated in an extensively glycosylated region of the HIV gp120 protein, where several antibodies targeting this region have shown broader neutralization breadth.^24^, ^25^ However, it remains unclear whether a “silent face” epitope exists on the SARS‐CoV‐2 Spike‐NTD, what its antigenicity and immunogenicity are.

The NTD of SARS‐CoV‐2 frequently mutates due to emerging and persistent variants, highlighting its vulnerability as an antigenic site under ongoing selective pressure.^13^ Most characterized NTD‐neutralizing antibodies target the “supersite” in a similar recognition manner and are susceptible to emerging mutations.^8^, ^10^ “Non‐supersite” antibodies, although sporadically reported, exhibit weaker neutralizing potency due to their indirect neutralization mechanisms, unlike targeted‐RBD‐neutralizing antibodies that interfere with the RBD‐hACE2 interaction.^8^, ^9^ Identifying antibodies targeting novel epitopes with unique neutralizing mechanisms is crucial for advancing our understanding of the SARS‐CoV‐2 spike protein's neutralizing epitopes.

In this study, we characterize a cryptic epitope on the SARS‐CoV‐2 spike‐NTD, shielded by robust glycans (Asn17, Asn122, and Asn165), and term it the “silent face,” inspired by a similar concept from the HIV gp120 protein. We identify the NTD “silent face” targeted neutralizing antibody 3711, which directly interacts with Asn17‐linked and Asn165‐linked glycans. Antibody 3711 is the first reported NTD silent face antibody for SARS‐CoV‐2, indicating that glycosylated regions or glycan shields can induce rare neutralizing antibodies in coronavirus infections.

Serological analysis of COVID‐19 patients indicated a narrow population distribution of the 3711‐class antibody, likely due to low immunogenicity caused by glycan shielding. Cryo‐EM structure analysis revealed an unusual contact surface between the NTD and antibody 3711. Previously reported NTD‐targeting antibodies are proposed to restrict spike conformational changes and prevent interaction with auxiliary receptors, thereby inhibiting virus‐cell membrane fusion.^6^, ^14^ In contrast, antibody 3711 competes with hACE2 via steric hindrance to block further viral entry.

Furthermore, our data indicate that 3711 primarily inhibits virus entry at the pre‐fusion stage and inhibits cell‐cell fusion, demonstrating multiple neutralization mechanisms. Although the neutralization efficacy of 3711 against Delta and Omicron BA.2.3 variants is reduced, the NTD‐targeting antibody exhibits broad neutralization against the WT strain, Alpha, Beta, Eta, and Omicron BA.1.1 variants. Additionally, it effectively protects mice infected with SARS‐CoV‐2 WT, Beta, and Omicron BA.1.1.

To elucidate the relationship between SARS‐CoV‐2 evolution and 3711 escape, we analyzed mutations in the Delta and BA.2.3 NTD, identifying two hotspot mutations: T19R/T19I and DEL157/158. These mutations, distinct from those found in Alpha, Beta, Eta, and Omicron BA.1.1 variants, alter the antigenic surface of the NTD. The T19R/T19I mutation results in the loss of the Asn17‐linked glycan, impairing interaction with antibody 3711, whereas the DEL157/158 mutation reshapes the NTD antigenic surface, causing a clash between 3711 and the NTD.

Our findings underscore the NTD as a critical domain of SARS‐CoV‐2 vulnerable to host humoral immune responses and flexible evolution. Further analysis of antibody gene usage in antibody repertoire sequencing revealed that the existence and prevalence of the 3711‐class silent face antibody is rare among COVID‐19 patients. Enhancing antibody affinity maturation through somatic hypermutation (SHM) may facilitate the development of a broader neutralization profile for antibodies of the 3711 clonotype. One limitation is that 3711 can neutralize WT, Alpha, Beta, Eta, and Omicron BA.1.1, but not Delta, BA.2.3, BA.5, or JN.1, limiting its ability to provide broad‐spectrum neutralizing protection. Exploring more innovative neutralizing antibodies could present promising strategies to overcome these limitations and achieve broader protection against emerging variants.

In conclusion, our study provides the first characterization of an uncommon glycan‐shielded epitope on the SARS‐CoV‐2 spike‐NTD, termed the “silent face.” Unlike common NTD‐targeting antibodies, the spike‐NTD “silent face” antibody 3711 neutralizes by blocking the RBD‐hACE2 interaction through steric hindrance. This characterization enriches our understanding of the immunogenicity of the SARS‐CoV‐2 spike protein and elucidates epitope‐guided viral mutations evading specific antibodies.

## 4 MATERIALS AND METHODS

### 4.1 Human Subjects and Antibody Screening

COVID‐19 patients confirmed by SARS‐CoV‐2 real‐time PCR and hospitalized at the First Affiliated Hospital of Guangzhou Medical University were enrolled.^33^ B lymphocytes were immortalized using EBV as previously described for screening SARS‐CoV‐2 specific antibodies.^23^ VH and VL sequences from positive B‐cell cultures were cloned into AbVec2.0‐IGHG1 and AbVec1.1‐IGKC or AbVec1.1‐IGLC2‐Xhol, respectively. Antibody production utilized the Expi293F Expression System. AmMag Protein A magnetic beads and PD‐10 columns were employed for antibody purification and desalting following the manufacturer's instructions.

### 4.2 SARS‐CoV‐2 authentic virus

The SARS‐CoV‐2 variants, including WT, Alpha (B.1.1.7), Beta (B.1.351), Delta (B.1.617.2), Eta (B.1.525) and Omicron BA.1.1 and Omicron BA.2.3 were isolated from COVID‐19 patients and maintained at Guangzhou Customs District Technology Center BSL‐3 Laboratory. The SARS‐CoV‐2 Delta (B.1.617.2) strain was provided by the Guangdong Provincial Center for Disease Control and Prevention, China. Experiments involving authentic SARS‐CoV‐2 were conducted in the Guangzhou Customs District Technology Center BSL‐3 Laboratory.

### 4.3 SARS‐CoV‐2 challenge experiment

Five to six‐week‐old female BALB/c mice were purchased from Gem Pharmatech. All animal experiments were approved by the Institutional Animal Care and Use Committees of Affiliated First Hospital of Guangzhou Medical University. Recombinant adenoviral vectors expressing human ACE2 (Ad5‐hACE2, 2.5 × 10^8^ FFU) were used to transduce mice for infection with the SARS‐CoV‐2 WT strain (1 × 10^5^ FFU) and Delta strain (5 × 10^4^ FFU), while wild‐type BALB/c mice were used for infection with the Beta strain (5 × 10^4^ FFU) and Omicron BA.1.1 strain (1 × 10^5^ FFU).^29^, ^34^ Antibodies (10 mg/kg) were administered prophylactically or therapeutically 24 hours before or after SARS‐CoV‐2 challenge. Mice were euthanized 1, 2, or 3 days post‐challenge for viral titer determination. Lung tissues were collected for hematoxylin‐eosin staining 4 days post‐challenge, and body weight changes in Beta‐infected mice were monitored for up to 10 days post‐infection.

### 4.4 Protein expression and purification for Cryo‐EM

Protein for Cryo‐EM was prepared as previously described.^35^ The extracellular domain sequence of spike (S‐ECD) with proline mutations at residues 986 and 987 was cloned into the pCAG vector followed by T4 foldon and Flag tag. For S‐6p construction, additional proline substitutions were introduced at residues 817, 892, 899, and 942.^11^ HEK293F cells were transfected with pCAG‐spike using polyethylenimines (PEIs). After 96 hours of culture, cellular supernatant containing secreted S‐ECD proteins was collected and purified using anti‐FLAG M2 affinity resin (Sigma Aldrich). Subsequent to extensive washing, the protein of interest was eluted, enriched, and subjected to size‐exclusion chromatography (Superose 6 Increase 10/300 GL, GE Healthcare). The concentrated S‐ECD protein was co‐incubated with 3711 or 26434 at a 1:5 molar ratio for one hour, and the resulting complex was purified by size‐exclusion chromatography. Peak fractions of the complex were collected and concentrated to approximately 1.5 mg/mL for EM analysis.

### 4.5 Cryo‐EM sample preparation

Cryo‐EM sample preparation was performed as previously reported.^36^ Briefly, protein complex was placed on glow‐discharged holey carbon grids, which were blotted and flash‐frozen. The prepared grids were transferred to a Titan Krios operating at 300 kV equipped with Gatan K3 detector and GIF Quantum energy filter. AutoEMation was used for movie stacks automatically collection.^37^ For a total of 32 frames per stack, each stack with a total dose rate of approximately 50 e‐/Å^2^ was exposed for 2.56 s with an exposure time of 0.08 s per frame. Stacks were motion corrected with MotionCor2^38^ and binned two‐fold. Dose weighting was performed^39^ and the defocus values were estimated with Gctf.^40^

### 4.6 Model building and structure refinement

For model building of the complex of S‐ECD protein of SARS‐CoV‐2 with 3711 or 26,434 respectively, the atomic model of the S‐ECD in complex 4A8 (PDB ID: 7C2L) were used as a template.^41^ As antibody 4A8 a template, the Chainsaw^42^ models of 3711 or 26434 were acquired which was further manually adjusted Coot with manually checked.^43^ For prevent overfitting, Phenix was performed for structural refinement with secondary structure and geometry restraints. The refined model was compared against the other map. Statistics associated with data collection, 3D reconstruction and model building were summarized in Table S1.

### 4.7 Data processing

As described, Relion 3.0.6 was used for the automatically pick of particles for the complex of S‐ECD protein with antibody 3711 or 26,434. Selected particles via Relion 3.0.6 were subjected to heterogeneous refinement without symmetry using cryoSPARC in three cycles and nonuniform refinement with C1 symmetry.^44^ The reconstruction of the complex was adopted to 3D auto‐refinement and post‐processing in Relion. To acquire the high‐quality map of the interface between Spike protein and 3711 or 26,434, datasets of the complex of NTD protein and related antibody were applied for the focused refinement on sub‐complex of each NTD protein and antibody. The resolution was estimated with the gold‐standard Fourier shell correlation 0.143 criterion^45^ with high‐resolution noise substitution (Figures S1 and S2; Table S1).^46^

### 4.8 Cell‐cell fusion inhibition assay via crystal violet staining

The cell‐cell fusion inhibition assay was adapted from previous methods with modifications.^27^, ^47^ HEK293T cells were transfected with plasmids encoding SARS‐CoV‐2 spike by Lipofectamine 2000 for 48 hours at 37°C. Transfected cells were incubated with serially diluted antibody 3711 at 37°C for 1 hour, then mixed 1:1 with Huh‐7 cells for 24 hours at 37°C in 48‐well plates. After washing with DPBS and fixation with 4% paraformaldehyde, cells were stained with crystal violet for syncytium visualization. The CTL ImmunoSpot S6 Ultra analyzer (Cellular Technology Limited) was used for syncytium formation observation and image capture. Triplicate wells were analyzed for each treatment.

### 4.9 Cell‐cell fusion inhibition assay via fluorescence checking

HEK293T cells were co‐transfected with plasmids encoding SARS‐CoV‐2 Spike and pEGFP using Lipofectamine 2000 for 48 hours at 37°C. Transfected cells were incubated with serially diluted antibody for 1 hour, then mixed 1:1 with Huh‐7 cells for 24 hours at 37°C in 96‐well plates. Syncytium formation was examined using a fluorescence microscope (EVOS digital inverted microscope, Invitrogen). Triplicate wells were analyzed for each treatment.

### 4.10 Screening of virus escape mutations by using authentic virus passage under the pressure of antibodies

To screen for antibody‐specific escape mutations, authentic SARS‐CoV‐2 was passaged under increasing antibody concentrations followed by next‐generation sequencing.^47^ Virus was incubated with antibodies 3711 and 26,434 from Passage 1 to Passage 12 (P1 to P12) with triplicate samples. Cell supernatant was harvested post‐CPE confirmation (> 50%) for subsequent passages. Antibody concentration was escalated to 2 × IC_50_ from P4 to P6, 4 × IC_50_ from P7 to P9, and 6 × IC_50_ from P10 to P12. Viral RNA from P6 and P12 was extracted using the QIAamp viral RNA extraction kit (Qiagen) and sequenced on an Illumina NextSeq 550. Mutation frequencies were analyzed and presented using CLC Genomics Workbench v11.0 and GraphPad Prism.

### 4.11 Next‐generation sequencing of SARS‐CoV‐2 genome

Viral RNA from P6 and P12 was extracted using the QIAamp viral RNA extraction kit (Qiagen) and sequenced according to recommended protocols (Vision Medicals, VW204‐96). Sequencing reads were quality assessed, and PCR amplicons were purified for sequencing on an Illumina NextSeq 550 using the Wuhan‐Hu‐1 (NC_045512.2) reference sequence. Mutation frequencies were analyzed using CLC Genomics Workbench v11.0 and visualized in GraphPad Prism.

### 4.12 Digestion of Asparagine‐linked glycans on NTD protein

PNGase F (New England Biolabs, P0704S) was used for nearly complete deglycosylation of non‐denaturing NTD protein following manufacturer instructions. Briefly, 20 μg of NTD protein, 2 μL of GlycoBuffer 2 for PNGase F, and 5 μL of PNGase F (2500 units) in a 20 μL reaction were incubated at 37°C for 24 hours. Denaturing deglycosylation of NTD protein served as a control. The extent of deglycosylation was assessed by SDS‐PAGE gel mobility shifts.

### 4.13 Class analysis of NTD antibody in serum of COVID‐19 patient

NTD‐specific antibody sequences were synthesized and cloned into AbVec2.0‐IGHG1, AbVec1.1‐IGKC, or AbVec1.1‐IGLC2‐XhoI.^48^ A serum‐NTD‐antibody competition assay was performed to analyze the distribution of NTD‐specific antibodies in convalescent serum. Antibodies (3711, 4–8, and P008_056) were precoated on 96‐well ELISA plates overnight at 4°C. Horseradish peroxidase (HRP)‐labeled NTD protein was mixed with diluted convalescent serum at 37°C for 1 hour, transferred to the antibody‐coated plates, and incubated at 37°C for 30 minutes. Tetramethylbenzidine (TMB) solution was used for ELISA reaction visualization, and OD_450_ values were read using a BioTek microplate reader. Negative controls were set using OD_450_ values of HRP‐NTD binding to the respective selected antibodies.

### 4.14 Analysis of antibody gene usage in COVID‐19 patients

Publicly available datasets (PRJCA007067 and PRJCA00375) from 33 COVID‐19 patients were analyzed to determine antibody gene usage abundance.^32^ IgH sequences were analyzed using MIXCR v3.0.3,^49^ and normalized expression levels of representative IGHV3‐53 antibodies, NTD supersite antibodies, and NTD silent face antibody 3711 were plotted using R software (https://www.r‐project.org/).

### 4.15 Statistical analysis

Statistical analysis was conducted using GraphPad Prism 7.04. Kinetic curves of protein binding (ELISA) and neutralization assay were fitted using nonlinear regression (dose‐response—stimulation and dose‐response—inhibition, respectively). Differences between groups were analyzed using Student's t‐test, with *p*‐values < 0.05 considered statistically significant (*p ≤ 0.05, **p ≤ 0.01, ***p ≤ 0.001, ****p ≤ 0.0001). Values are presented as mean ± SEM.

## AUTHOR CONTRIBUTIONS

Jincun Zhao, Qiang Zhou, Yanqun Wang, and Jingxian Zhao––initiated and coordinated the project. Zhaoyong Zhang, Yanqun Wang, and Lu Zhang––evaluated the neutralizing potency using authentic virus in vitro and vivo. Yuanyuan Zhang, Yuting Zhang, and Yingying Guo––performed cryo‐EM studies. Yanqun Wang, and Zhaoyong Zhang––designed the experiments and wrote the manuscript. Yimin Li, Lei Chen, Xuesong Liu, Xiaoqing Liu, Yingkang Jin, and Yonghao Xu––revised the manuscript. Yuting Zhang, Lu Zhang, Jun Dai, and Jiantao Chen––performed the affinity experiments. Peilan Wei, Xinyi Xiong, Shengnan Zhang, Juxue Xiao, Airu Zhu, Jianfen Zhuo, Ruoxi Cai, Jingjun Zhang, Haiyue Rao, Yuanyuan Zhang, Lei Chen and Bin Qu––evaluated the neutralizing potency using pseudovirus and performed escape mutant screening under pressure of antibody. Qihong Yan, Jiaxin Feng, Jinling Cheng, Jingyi Su, Canjie Chen, and Shu Li––participated in data analysis. All authors have read and approved the final manuscript.

## CONFLICT OF INTEREST STATEMENT

The authors declare no conflicts of interest.

## ETHICS STATEMENT

This study was approved by the Ethics Committee of The First Affiliated Hospital of Guangzhou Medical University (2022030). Written informed consent was obtained from all participants. The animal study was approved by the Institutional Animal Care and Use Committees of Affiliated First Hospital of Guangzhou Medical University (20230616).

## Supporting information



Supporting Information

## Data Availability

The data supporting the results in this study are available from the corresponding author upon reasonable request. Atomic coordinates and cryo‐EM density maps of the S protein of SARS‐CoV‐2 in complex with 3711 (PDB: 8HLC; whole map: EMD‐34872, antibody‐epitope interface‐focused refined map: EMD‐34873) and 26434 (PDB: 8HLD; whole map: EMD‐34874, antibody‐epitope interface‐focused refined map: EMD‐34875) have been deposited to the Protein Data Bank (http://www.rcsb.org) and the Electron Microscopy Data Bank (https://www.ebi.ac.uk/pdbe/emdb/), respectively.
