# Supplementary material for: Defining the features and structure of neutralizing antibody targeting the silent face of the SARS‐CoV‐2 spike N‐terminal domain
Source: MedComm (2020). 2024 Nov 28;5(12):e70008. doi: 10.1002/mco2.70008 (PMC11604724; doi:10.1002/mco2.70008)
Supplement: Supplementary file 1 — Supporting Information [file MCO2-5-e70008-s001.docx]

Supplementary Materials

**Defining the Features and Structure of Neutralizing Antibody Targeting the Silent Face of the SARS-CoV-2 Spike N-terminal Domain**

Zhaoyong Zhang^1,#^, Yuanyuan Zhang^2,#^, Yuting Zhang^1,#^, Linling Cheng^1,#^, Lu Zhang^3^, Qihong Yan^1^, Xuesong Liu^1^, Jiantao Chen^1^, Jun Dai^3^, Yingying Guo^4^, Peilan Wei^1,5^, Xinyi Xiong^6^, Juxue Xiao^1^, Airu Zhu^1^, Jianfen Zhuo^1^, Ruoxi Cai^1^, Jingjun Zhang^1^, Haiyue Rao^1^, Bin Qu^6^, Shengnan Zhang^1^, Jiaxin Feng^1^, Jinling Cheng^1^, Jingyi Su^1^, Canjie Chen^1^, Shu Li^1^, Yuanyuan Zhang^1^, Lei Chen^1^, Yingkang Jin^7^, Yonghao Xu^1^, Xiaoqing Liu^1^, Yimin Li^1^, Jingxian Zhao^1,5,*^, Yanqun Wang^1,8,9*^, Qiang Zhou^2,*^, Jincun Zhao^1,5,6,10*^

^1^State Key Laboratory of Respiratory Disease, National Clinical Research Center for Respiratory Disease, Guangzhou Institute of Respiratory Health, the First Affiliated Hospital of Guangzhou Medical University, Guangzhou, China

^2^Center for Infectious Disease Research, Research Center for Industries of the Future, Zhejiang Key Laboratory of Structural Biology, School of Life Sciences, Westlake University; Institute of Biology, Westlake Institute for Advanced Study; Westlake Laboratory of Life Sciences and Biomedicine, Hangzhou, Zhejiang Province, China

^3^Health and Quarantine Laboratory, Guangzhou Customs District Technology Centre, Guangzhou, China

^4^Southern University of Science and Technology, Shenzhen, China

^5^Guangzhou National Laboratory, Bio-Island, Guangzhou, China

^6^Shanghai Institute for Advanced Immunochemical Studies, School of Life Science and Technology, ShanghaiTech University, Shanghai, China

^7^Pediatric Pulmonary Department, Guangzhou Women and Children's Medical Center, Guangzhou Medical University, Guangzhou, China

^8^Clinical Laboratory Medicine Department, The Second Affiliated Hospital of Guangzhou Medical University, Guangzhou, China

^9^GMU-GIBH Joint School of Life Sciences, Guangzhou Medical University, Guangzhou, China

^10^Institute for Hepatology, National Clinical Research Center for Infectious Disease, Shenzhen Third People’s Hospital; The Second Affiliated Hospital, School of Medicine, Southern University of Science and Technology, Shenzhen, China

***Correspondence**

Jincun Zhao, Yanqun Wang, and Jingxian Zhao, State Key Laboratory of Respiratory Disease, National Clinical Research Center for Respiratory Disease, Guangzhou Institute of Respiratory Health, the First Affiliated Hospital of Guangzhou Medical University, 195 Dongfengxi Road, Guangzhou 510000, China.

Email: zhaojincun@gird.cn; [wangyanqun@gird.cn](mailto:wangyanqun@gird.cn); [zhaojingxian@gird.cn](mailto:zhaojingxian@gird.cn)

Qiang Zhou, Key Laboratory of Structural Biology of Zhejiang Province, Institute of Biology, Westlake Institute for Advanced Study, School of Life Sciences, Westlake University, 600 Dunyu Road, Hangzhou 310030, China.

Email: zhouqiang@westlake.edu.cn

^#^Zhaoyong Zhang, Yuanyuan Zhang, Yuting Zhang and Linling Cheng contributed equally to this work.

This PDF file includes:

**Materials and methods**

**Supplementary Figure S1 to S8**

**Supplementary Table S1 to S2**

**Materials and methods**

**Protein binding assay**

SARS-CoV-2 Spike (S1+S2 ectodomain) and NTD protein were purchased from Sino Biological (40589-V08B1) and Novoprotein (DRA45), respectively. Twenty-five micrograms of protein were coated in the 96-well ELISA plate overnight. Plates were blocked with 10% fetal bovine serum (FBS) for two hours, and then serially diluted antibodies were added as first antibody for two hours. Peroxidase conjugated AffiniPure Goat Anti-Human IgG (H+L) antibody (Jackson ImmunoResearch, 109-035-088) and TMB substrate were used for reaction visualization. Values of OD450 were read by BioTek microplate reader. Kinetics graphs were composed in GraphPad Prism.

**Binding affinity via biolayer interferometry assay**

Biolayer interferometry assay (BLI) was conducted for the test of affinity of antigen-antibody binding using Octet RED96 (SARTORIUS). Octet SA (Streptavidin) Biosensors (SARTORIUS, 18-5020) was used for Spike protein (200 nM, 100 nM, 50 nM, 25 nM, 12.5 nM, 6.25 nM, 3.125 nM) immobilization, the concentration of antibody was set to 11 μg/mL (73.3 nM). Briefly, binding affinity test was performed as below: (1) Kinetics baseline with DPBS: 60s; (2) Antigen Loading: 25s; (3) Baseline with DPBS: 60s; (4) Antibody association: 300s; (5) Antibody disassociation: 600s; (6) Regeneration and Neutralization: 30s. The data was analyzed in Fortebio Date Analysis Software (Version: 9.0.0.6), dissociation constant values (KD) were acquired by 1:1 fitting model, and Kinetics graphs were composed in GraphPad Prism.

**Antibody-hACE2 competition analysis using biolayer interferometry**

SARS-CoV-2 Spike protein (40 μg/ml, 283.7 nM) was first immobilized on Octet Streptavidin (SA) Biosensor (SARTORIUS, 18-5019), and then 400 nM of hACE2 and 3711 was loaded sequentially for binding and competition. Briefly, the competition program was set as below: (1) Kinetics baseline with DPBS: 60s; (2) Antigen Loading: 300s; (3) Baseline with DPBS: 60s; (4) First antibody (protein) association: 300s; (5) Second antibody (protein) association: 300s; (6) Regeneration and Neutralization: 30s. The assay of Spike-ACE2-ACE2, Spike-ACE2-Buffer, Spike-Buffer-Buffer, Spike-3711-3711, Spike-3711-ACE2, and Spike-3711-Buffer were performed in the same setting. The data was analyzed in Fortebio Date Analysis Software (Version: 9.0.0.6), and Kinetics graphs were composed in GraphPad Prism.

**Antibody-hACE2 competition analysis using flow cytometry**

HEK293T cells were transfected with SARS-CoV-2 Spike plasmid (pCDNA3.1-SARS-CoV-2-Spike) via lipofectamine 2000 (Thermo). Cells expressing SARS-CoV-2 Spike were harvested after 48 hours and incubated with serially diluted antibody 3711 and 26434 (500 μg/mL, 200μg/mL, 100 μg/mL, 20 μg/mL, 4 μg/mL, 0.8 μg/mL) for 2 hours at 4 °C. After washed twice by washing buffer (DPBS + 5 % FBS), cells were incubated with the 20 μg/mL of protein of ACE2 fused with mouse immunoglobulin fragment (ACE2-mIg) for 30 minutes at 4 °C and then stained with Alexa Fluor® 647 AffiniPure Goat Anti-Mouse IgG (H+L) (Jackson immunoresearch, 115-605-062) for 15 minutes at 4 °C. Cells with triplicates in same treatment were analyzed by FACS Verse instrument (BD Bioscience). The percentage of ACE2 binding was corresponded to the A647 Goat Anti-Mouse IgG (H+L) positive rate. The positive rate in the control with Spike-expressing cell incubated with ACE2-mIg was adjusted to 100%. Dates were plotted in GraphPad Prism.

**Virus titer determination by focus-forming assay (FFA)**

FFA was used to detect the virus quantification of SARS-CoV-2 authentic virus and lung homogenate of mice. The SARS-CoV-2 strains isolated from COVID-19 patients were restored in Guangzhou Customs District Technology Center BSL-3 Laboratory. Samples to be tested were serially diluted and added to pre-seeded Vero E6 cells in 96-well plate for one-hour incubation. Then cells were covered with Minimum Essential Medium (MEM) comprising 1.2% Carboxymethylcellulose (CMC) for 24 hours for focus forming. After fixed by 4% paraformaldehyde, cell plates were treated and stained with 0.2% Triton X-100, SARS-CoV/SARS-CoV-2 Nucleocapsid Rabbit PAb (Sino Biological, 40143-T62) and Peroxidase AffiniPure Goat Anti-Rabbit IgG (H+L) (Jackson immunoresearch, 111-035-144) for foci visualization. CTL ImmunoSpot S6 Ultra analyzer (Cellular Technology Limited) was used to foci analysis and titer determination.

**Authentic virus neutralization assay**

Focus reduction neutralization test (FRNT) was conducted for testing neutralization capacity of antibody and serum. Antibody and serum were first co-incubated with virus quantified by FFA. The mixtures were transferred to cell plate pre-seeded with Vero E6 for virus entry. The following steps were performed as FFA method.

**Authentic virus pre-attachment and post-attachment assay**

For pre-attachment assay, serially diluted antibodies were co-incubated with 100 FFU of SARS-CoV-2 for 1h at 4 °C, and then mixtures were transferred to cell plates (Greiner Bio-one, 655090) pre-seeded with Vero E6 cell for the other one hour-incubation at 4 °C for cell attachment. Then, after incubating at 37 °C for 30 min for entry, cells were overlaid with DMEM with 2% FBS. For post-attachment assay, 200 FFU of SARS-CoV-2 was transferred to Vero E6 cell plate for 1h at 4 °C for attachment. Virus attached cells were co-incubated with serially diluted antibodies for for 1h at 4 °C for interaction and for 30 min at 37 °C for virus entry. Then mixtures were dumped, and overlaid with DMEM with 2% FBS. After 24-hour incubation at 37 °C, cell plates of pre-attachment and post-attachment assay were fixed with 4% PFA and conducted to immunofluorescence assay (IFA). After permeabilized and blocked with 0.2 % Triton in 1 % Bovine Serum Albumin (BSA), cell plates were incubated with SARS-CoV/SARS-CoV-2 Nucleocapsid Rabbit PAb (Sino Biological, 40143-T62) and Alexa Fluor 488 AffiniPure Donkey Anti-Rabbit IgG(H+L) (Jackson immunoresearch, 711-545-152) in sequence. DAPI was used for nuclei visualization, SARS-CoV-2 specific signal was read and analyzed by Imaging Cytometer (Nexcelom Celigo).

**Protein expression and purification for binding assay (BLI and ELISA)**

Briefly, DNA sequence of the ectodomain of Spike (2019-nCoV BetaCoV/Wuhan/WIV04/2019, GISAID accession no. EPI_ISL_402124) was synthesized and subcloned into a pCDNA3.1+ plasmid vector. For trimeric spike stabilized construction, residues 682-685 (RRAR) have been deleted at the S1/S2 cleavage site and replaced with a single arginine to re-resemble the cleavage site. S-R production and purification were conducted as previously described. For NTD protein expression, sequence of SARS-CoV-2 NTD (NCBI Reference Sequence: NC_045512.2) with an N-terminal signal peptide and an N-terminal His tag was synthesized and subcloned into a pCDNA3.1^+^ plasmid vector. NTD protein was expressed by Gibco HEK Expi293 Expression system (ThermoFisher Scientific) by transient transfection using ExpiFectamine 293 reagent. Targeted protein was purified from cell supernatant by Ni-NTA Agarose (Qiagen) and eluted by 100 mM imidazole, then was assessed by mobility on SDS-PAGE gel. Moreover, targeted protein was ultrafiltrated by centrifugal filter devices (Amicon Ultra 10K device, Millpore) for desalting and buffer exchanging.


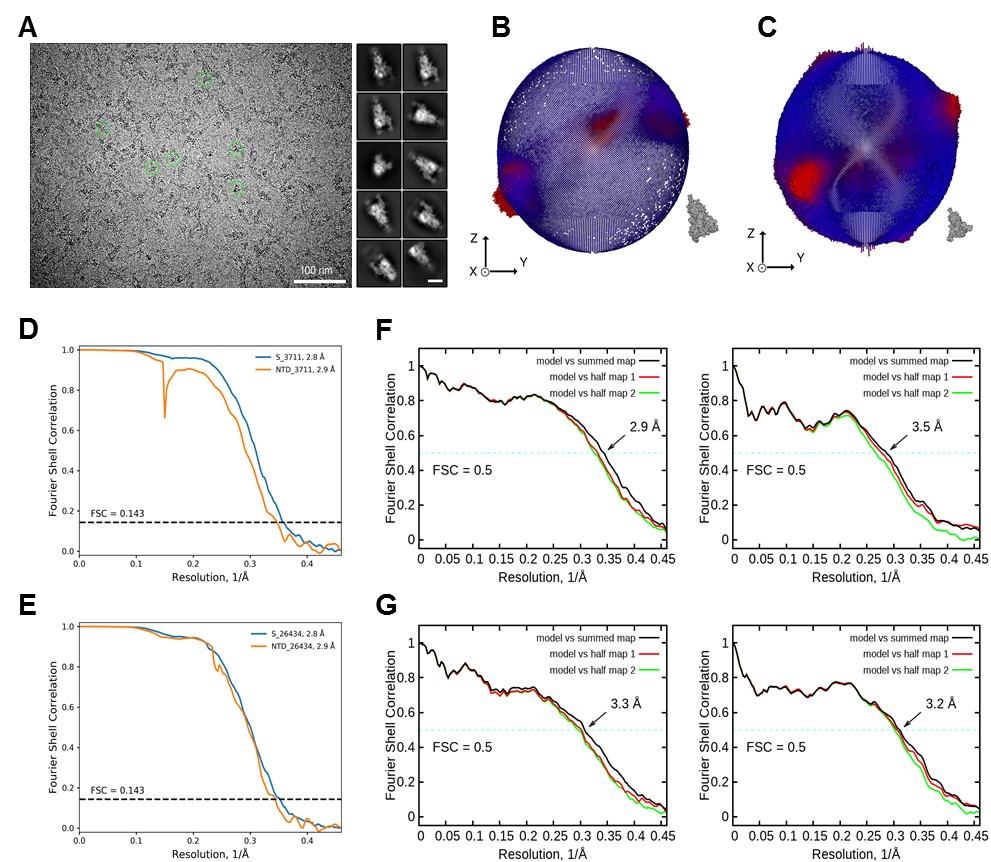


**Figure S1. Cryo-EM analysis of S-ECD in complex with 3711 or 26434.**

**(A)** Representative cryo-EM micrograph (magnification of 81,000x) and 2D class averages of cryo-EM particle images of S-ECD bound with 3711. The scale bar in 2D class averages is 10 nm.

**(B and C)** Euler angle distribution in the final 3D reconstruction of S-ECD bound with 3711 or 26434, respectively.

**(D and E)** FSC curve of the overall structure (blue) and NTD-Ab sub-complex (orange) of S-ECD bound with 3711 or 26434, respectively.

**(F and G)** FSC curve of the refined model of S-ECD bound with 3711 or 26434 versus the overall structure that it is refined against (black); of the model refined against the first half map versus the same map (red); and of the model refined against the first half map versus the second half map (green). The small difference between the red and green curves indicates that the refinement of the atomic coordinates did not suffer from overfitting.


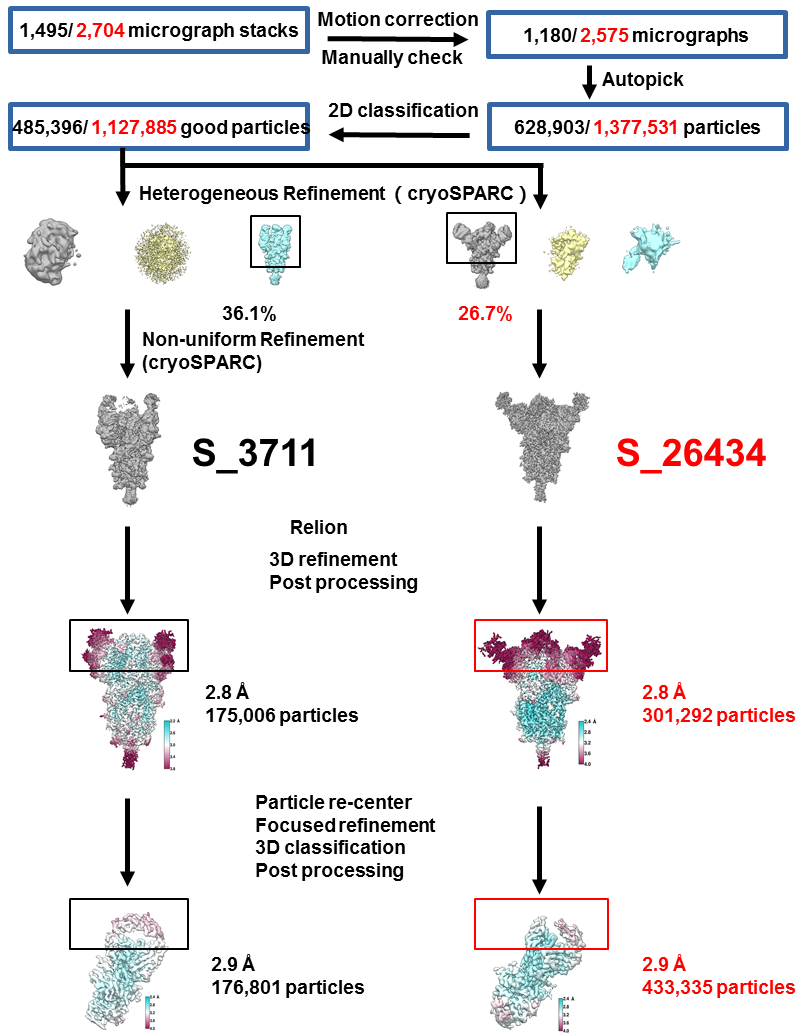


**Figure S2. Flowchart for cryo-EM data processing.**

Please refer to the ‘Data Processing’ in Methods section for details.


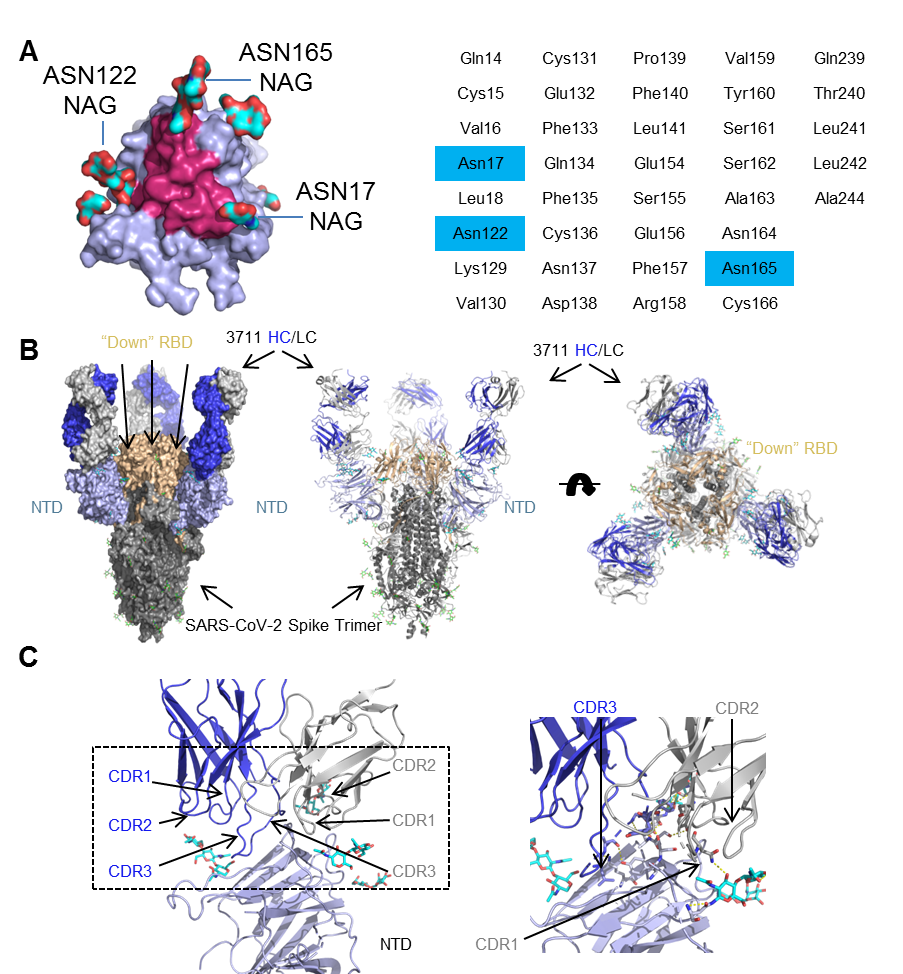


**Figure S3. Footprint of NTD silent face, cryo-EM structure of the complex of 3711 and S-ECD and the interaction between 3711 and NTD**

**(A)** Footprint and residues composition of NTD silent face. Asparagine linked glycans were colored cyan and red in the type of surface, residues on silent face were colored as hot pink. Residues composed NTD silent face were list on the right with Asn17, Asn122 and Asn165 colored with blue background.

**(B)** Cryo-EM structure of the 3711 and S-ECD complex. The surface map of complex is shown on the left, and two perpendicular views of the complex in the type of cartoon were shown on the right. Three NTDs of the trimeric S protein are colored lightblue, while three “closed” RBD are colored wheat. The heavy chain and light chain of 3711 are colored darkblue and gray, respectively.

**(C)** The relative spatial position of NTD and 3711 interaction. Only one NTD and 3711 are shown.


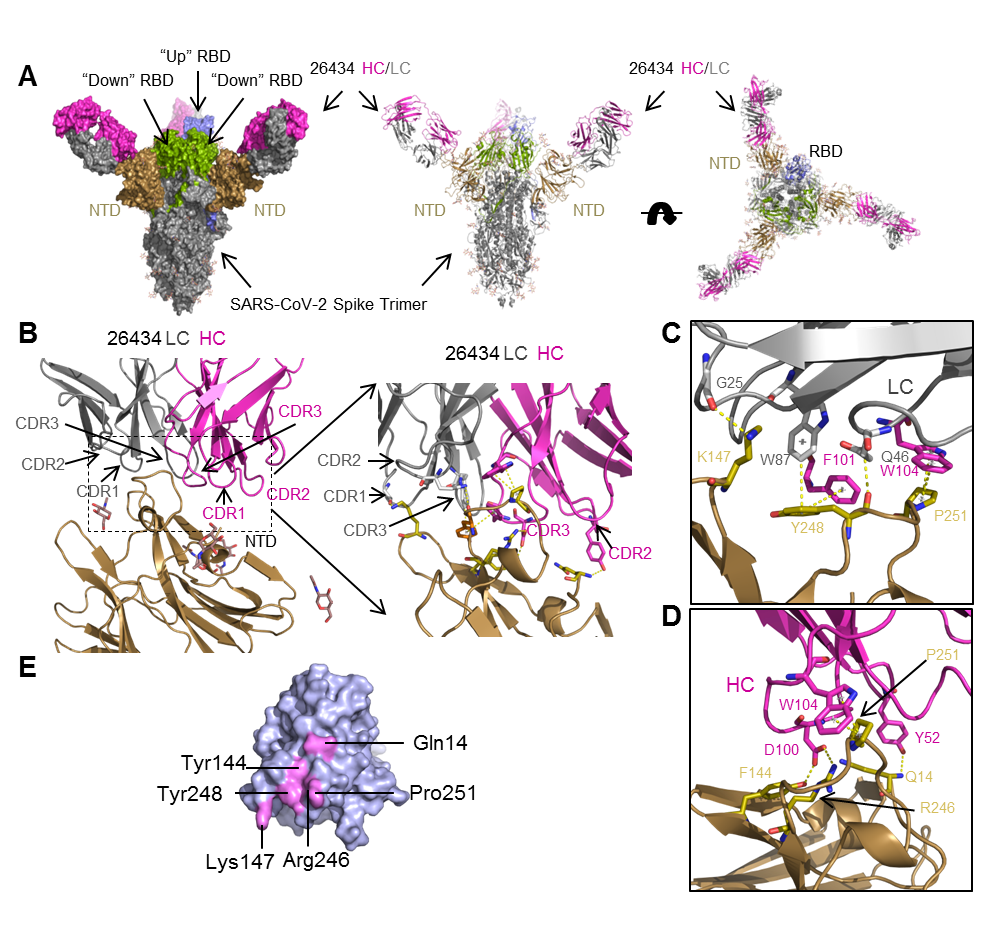


**Figure S4. Cryo-EM structure of the complex of 26434 and S-ECD and the interaction between 26434 and NTD**

**(A)** Cryo-EM structure of the 26434 and S-ECD complex. The surface map of complex is shown on the left, and two perpendicular views of the complex in the type of cartoon were shown on the right. Three NTDs of the trimeric S protein are colored darkyellow, while the “open” RBD and “closed” RBD are colored blue and green, respectively. The heavy chain and light chain of 26434 are colored magenta and gray, respectively.

**(B)** The relative spatial position of the identification of NTD and 26434. Only one NTD and 3711 are shown. The heavy chain and light chain of 26434 are colored magenta and gray, respectively. NTD is colored orange.

**(C)** and **(D)** detailed interactions between the NTD and 26434 light chain. Residues involved in interaction are presented in the type of stick. Polar interactions are indicated by yellow dashed lines. The heavy chain and light chain of 26434 are colored magenta and gray, respectively.

**(E)** The footprint of 26434 on NTD. Residues on NTD interacting with 26434 are colored pink.


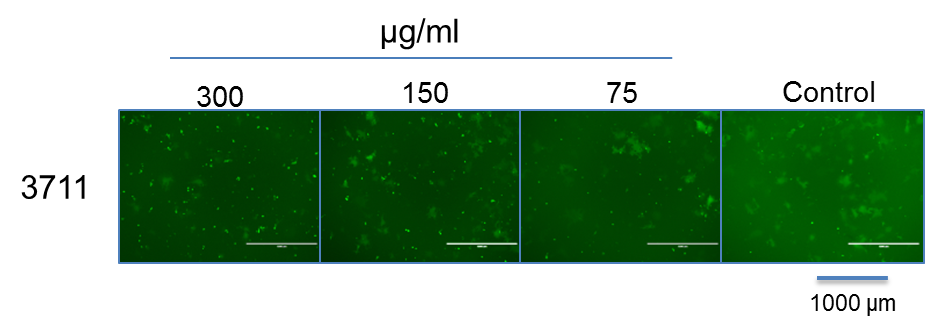


**Figure S5. 3711 inhibits cell-cell fusion**

Cell-cell fusion inhibition capacity of antibody 3711. HEK-293T cells expressing GFP and SARS-CoV-2 Spike were conducted to incubate with serially diluted antibody 3711, and then added to HuH-7 cells for further fusion. HEK-293T cells expressing GFP and SARS-CoV-2 Spike without incubating with antibody worked as a fusion control.


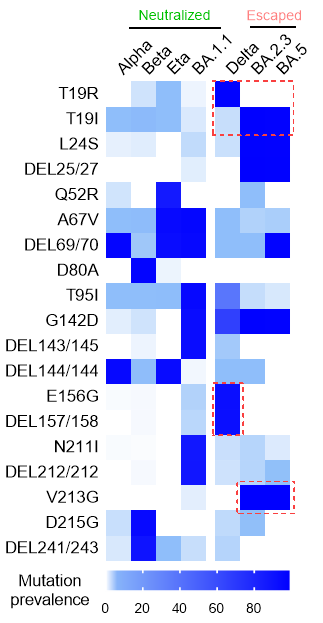


**Figure S6. Heatmap of specific mutation site on NTD protein of SARS-CoV-2 variants based on GISAID dataset**. Variants were divided to “Neutralized” and “Escaped”. Mutation hotspots were indicated as red dotted boxes


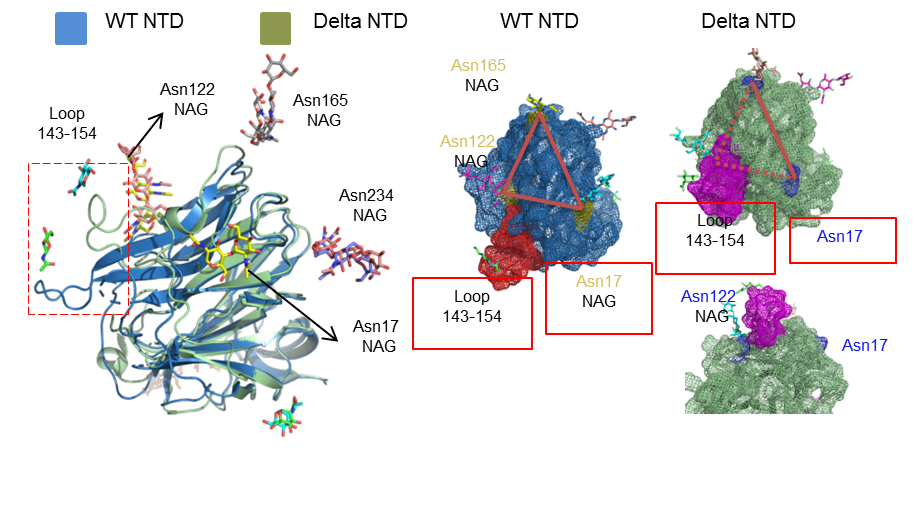


**Figure S7. The “Silent Face” was destructed due to the structure alteration of Loop 143-154 on Delta NTD.** The superposed model of Delta NTD (Olive, PDB: 7SBK) and WT NTD (Blue, PDB: 6VXX) in cartoon type was presented on the left. Loop 143-154 was indicated as red dotted box. WT NTD in mesh type was presented on the middle (Blue), with Loop 143-153 was indicated in red and silent face was indicated in a red triangle (Residues Asn17, Asn122 and Asn165 were indicated in yellow). Delta NTD in mesh type was presented on the right (Olive), with Loop 143-153 was indicated in magenta, but silent face was sheltered (Residues Asn17, Asn122 and Asn165 were indicated in blue).


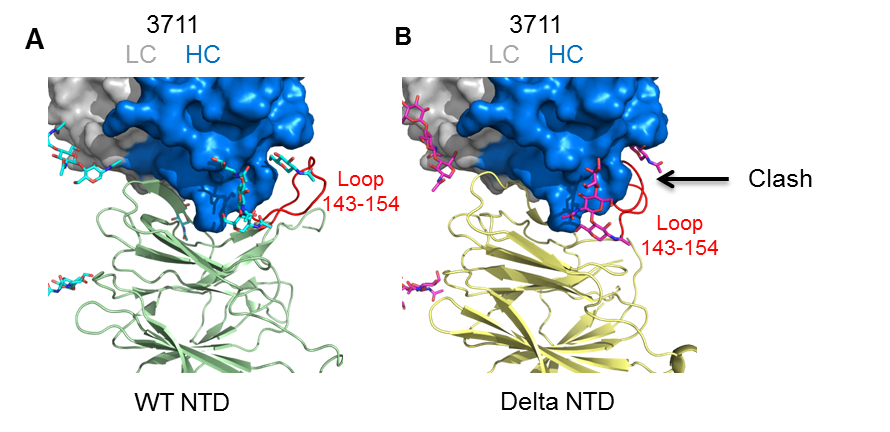


**Figure S8. The superposed model of 3711 with WT NTD and Delta NTD.**

**(A)** The overall conformation of the complex of WT NTD and 3711. The heavy chain and light chain of 3711 presented as surface type are colored blue and gray, respectively. WT NTD is colored green. Loop 143-154 is indicated in red.

**(B)** The overall conformation of the complex of Delta NTD and 3711. Delta NTD is colored yellow. Loop 143-154 is indicated in red. The clash between NTD and 3711 is indicated by a black arrow.

**Table.S1. Data collection, 3D reconstruction and model statistic.**

| **Data collection** |  | | | |
| --- | --- | --- | --- | --- |
| EM equipment | Titan Krios (Thermo Fisher Scientific) | | | |
| Voltage (kV) | 300 | | | |
| Detector | Gatan K3 Summit | | | |
| Energy filter | Gatan GIF Quantum, 20 eV slit | | | |
| Pixel size (Å) | 1.087 | | | |
| Electron dose (e-/Å2) | 50 | | | |
| Defocus range (μm) | -1.2 ~ -2.2 | | | |
| Sample | S-ECD_3711 | | S-ECD_26434 | |
| Number of collected micrographs | 1,495 | | 2,704 | |
| Number of selected micrographs | 1,180 | | 2,575 | |
| **3D Reconstruction** |  |  |  |  |
| Software | Relion 3.0 | | | |
| Sample | Overall | sub-complex | Overall | sub-complex |
| Number of used particles (Overall) | 175,006 | 176,801 | 301,292 | 433,335 |
| Resolution (Å) | 2.8 | 2.9 | 2.8 | 2.9 |
| Symmetry | C1 | | | |
| Map sharpening B-factor (Å2) | -90 | | | |
| **Refinement** |  | | | |
| Software | Phenix | | | |
| Cell dimensions |  | | | |
| a=b=c (Å) | 313.056 | | | |
| α=β=γ (˚) | 90 | | | |
| Model composition |  | | | |
| Protein residues | 4,569 | | 4,507 | |
| Side chains assigned | 4,569 | | 4,507 | |
| Sugar | 93 | | 87 | |
| Linoleic acid | 3 | | 0 | |
| R.m.s deviations |  | | | |
| Bonds length (Å) | 0.010 | | 0.007 | |
| Bonds Angle (˚) | 0.880 | | 0.839 | |
| Ramachandran plot statistics (%) |  | | | |
| Preferred | 94.85 | | 93.73 | |
| Allowed | 5.06 | | 6.17 | |
| Outlier | 0.09 | | 0.10 | |

**Table.S2. Gene usage of enrolled NTD antibodies.**

| **ID** | **V_H_** | | | **V_κ/λ_** | | | **PDB** |
| --- | --- | --- | --- | --- | --- | --- | --- |
|  | **V Gene** | **J Gene** | **CDR3 (aa)** | **V Gene** | **J Gene** | **CDR3 (aa)** |  |
| **3711** | V3-11 | J6 | ARGGWELRSLAGGYYGMDV | KV1-17 | KJ1 | LQHNSYPWT | - |
| **5-7** | V1-46 | J6 | ARDREPHSDSSGYWDSLKYYYYYALDV | KV1-9 | KJ3 | QQLNTYPFT | 7RW2 |
| **DH1052** | V1-69-2 | J4 | ATSSGPSRLCGGGSCYHSFDY | KV3-20 | KJ1 | QQYGSSPTWT | 7LAB |
| **P008_056** | V3-21 | J4 | ASNRSPYDSSNYYFDY | KV1-33 | KJ4 | QHHDSLPLT | 7NTC |
| **26434** | V1-18 | J5 | ARSPPDFLGWFDP | LV3-1 | LJ2 | QAWDSSTVI | - |
| **4-8** | V1-69 | J6 | ASLQTVDTAIEKYYGMDV | LV3-1 | LJ3 | QAWDSSTAV | 7LQV |

In addition to antibodies 3711 and 26434, previously reported antibodies 5-7, DH1052, P008_056 and 4-8 were also included as NTD antibody controls. Sequences are analyzed using IMGT.
